# Fob1-dependent condensin recruitment and loop extrusion on yeast chromosome III

**DOI:** 10.1371/journal.pgen.1010705

**Published:** 2023-04-14

**Authors:** Manikarna Dinda, Ryan D. Fine, Shekhar Saha, Zhenjia Wang, Chongzhi Zang, Mingguang Li, Jeffrey S. Smith

**Affiliations:** 1 Department of Biochemistry and Molecular Genetics, University of Virginia School of Medicine, Charlottesville, Virginia, United States of America; 2 Department of Microbiology, Immunology, and Cancer Biology, University of Virginia School of Medicine, Charlottesville, Virginia, United States of America; 3 Center for Public Health Genomics, University of Virginia School of Medicine, Charlottesville, Virginia, United States of America; 4 Department of Laboratory Medicine, Jilin Medical University, Jilin, China; Netherlands Cancer Institute, NETHERLANDS

## Abstract

Despite recent advances in single-molecule and structural analysis of condensin activity *in vitro*, mechanisms of functional condensin loading and loop extrusion that lead to specific chromosomal organization remain unclear. In *Saccharomyces cerevisiae*, the most prominent condensin loading site is the rDNA locus on chromosome XII, but its repetitiveness deters rigorous analysis of individual genes. An equally prominent non-rDNA condensin site is located on chromosome III (chrIII). It lies in the promoter of a putative non-coding RNA gene called *RDT1*, which is in a segment of the recombination enhancer (RE) that dictates *MAT*a-specific chrIII organization. Here, we unexpectedly find that condensin is recruited to the *RDT1* promoter in *MAT*a cells through hierarchical interactions with Fob1, Tof2, and cohibin (Lrs4/Csm1), a set of nucleolar factors that also recruit condensin to the rDNA. Fob1 directly binds to this locus *in vitro*, while its binding *in vivo* depends on an adjacent Mcm1/α2 binding site that provides *MAT*a cell specificity. We also uncover evidence for condensin-driven loop extrusion anchored by Fob1 and cohibin at *RDT1* that unidirectionally extends toward *MAT*a on the right arm of chrIII, supporting donor preference during mating-type switching. *S*. *cerevisiae* chrIII therefore provides a new platform for the study of programmed condensin-mediated chromosome conformation.

## Author summary

Condensin is a conserved protein complex that compacts chromosomes during mitosis through a combination of protein-protein interactions and DNA loop extrusion. There is active discussion regarding the mechanisms of condensin loading onto chromatin and directionality of the loop extrusion process, which may be organism specific. In budding yeast, the single condensin complex performs one-sided loop extrusion *in vitro*, but there are no examples of specific condensin recruitment sites where loop extrusion can be mechanistically studied *in vivo*. We previously identified a novel condensin recruitment site on chromosome III at the recombination enhancer locus, which controls structural conformation *in cis*. We now report the mechanism of condensin recruitment onto chromosome III, discovering it occurs through a complex of ‘nucleolar’ proteins anchored onto chromatin by Fob1, also known to recruit condensin onto the ribosomal DNA. On chromosome III, Fob1 cooperates with a MADS-box transcription factor, Mcm1, to specifically recruit condensin in *MAT*a cells, thus providing cell type regulation. Furthermore, we observed evidence of condensin-mediated loop extrusion anchored by Fob1 on chromosome III. In the future, yeast chromosome III should provide a powerful genetic tool for understanding regulatable condensin loading and loop extrusion that organizes chromosome organization from a single site *in vivo*.

## Introduction

Condensins are highly conserved multi-subunit complexes that function in chromosome organization and segregation. In budding yeast, *Saccharomyces cerevisiae*, the single condensin complex consists of two Structural Maintenance of Chromosome (SMC) ATPase subunits (Smc2 and Smc4), and three regulatory non-SMC subunits (Ycs4, Ycg1 and Brn1). Brn1 serves as a kleisin subunit that bridges the ATP-binding domains of Smc2 and Smc4 [1]. As with other SMC complexes, yeast condensin can form DNA loops through an ATP-dependent loop-extrusion activity [2]. Condensin has also been hypothesized to self-associate to bring distant chromatin-bound loci into contact [3].

A general mechanism for loading mitotic condensin I and II onto chromatin remains elusive, especially for vertebrates that must extensively condense their large genomes and may require relatively non-specific recruitment. More is known about specific condensin recruitment during interphase with invertebrates, including the *C*. *elegans* dosage compensation complex (DCC), a specialized condensin that reduces gene expression from the two hermaphrodite X-chromosomes by half (reviewed in [4]). DCC is recruited to a set of primary recruitment sites on the X-chromosome by several non-condensin factors, including SDC-2 and SDC-3 [5]. SDC-2 also maintains some of these strong recruitment sites in an open chromatin conformation [5]. Condensin complexes in *S*. *cerevisiae* and *S*. *pombe*, which have much smaller genomes, are attracted to transcriptionally active chromatin regions through interactions with transcription factors and enhanced by nucleosome displacement [6,7]. At tRNA genes in *S*. *cerevisiae*, for example, condensin is recruited by association with RNA Pol III transcription factors and the Scc2/Scc4 cohesin loading complex, where it mediates tRNA gene clustering into several foci surrounding the nucleolus [8]. An emerging theme for these forms of specific condensin recruitment involves opening of chromatin.

The predominant condensin target in *S*. *cerevisiae* is the repetitive rDNA tandem array located on the right arm of chromosome XII (chrXII), around which the nucleolus forms. Condensin is specifically recruited to the intergenic spacer region (IGS1) through association with a nucleolar protein complex established by the DNA replication fork block protein Fob1 [9]. Fob1 directly binds to cis-acting DNA sequences (TER1 and TER2) within IGS1 [10,11], unidirectionally blocking replication fork progression to prevent head-on collisions with RNA polymerase I [12,13]. Collapse of the stalled forks leads to DNA double-strand breaks (DSB) [14], and oligomerization of Fob1 brings distant TER sites into proximity through ‘chromosome kissing’ to facilitate repair through homologous recombination [15]. Unequal sister chromatid exchange during DSB repair produces extrachromosomal rDNA circles (ERCs) that accumulate in mother cells and negatively impact replicative lifespan [16,17]. Deletion of *FOB1* prevents the fork blocks and DSB formation, which suppresses ERC accumulation and extends lifespan [18].

Fob1 and its associated factors, cohibin (Lrs4, Csm1) and Tof2, also recruit cohesin and the nucleolar Sir2 histone deacetylase (HDAC) complex, RENT, to the rDNA [19]. RENT recruitment mediates transcriptional silencing of intergenic non-coding RNAs [20,21], and also regulates exit from mitosis as a component of the FEAR network [22,23]. Cohibin is a mitotic version of the meiotic monopolin complex [24,25], which forms a V-shaped coiled-coil structure that crosslinks kinetochore components and appears to tether rDNA repeats to the nuclear periphery [26]. Unlike cohibin, Fob1 has not previously been implicated in any functions outside the rDNA locus.

Another major condensin recruitment site in *S*. *cerevisiae* is specific to *MAT*a cells, occurring at the recombination enhancer (RE) located approximately 16 kb away from the silent mating-type locus, *HML*, on the left arm of chromosome III (chrIII) [27,28]. The other silent mating-type locus, *HMR*, is located on the right arm of chrIII. The RE is required for mating-type switching donor preference in *MAT*a cells, whereby the DSB induced by HO endonuclease at *MAT*a is repaired by homologous recombination using *HML* as the donor template, thus ensuring conversion from *MAT*a to *MAT*α (reviewed in [29]). The RE is a composite element with the left half required for donor preference activity [28,30], and the right half required for establishing a specialized chrIII structure that positions *HML* in proximity with the centromere and *MAT*a locus [31], and also limits association of *HMR* with *MAT*a [27]. Condensin recruitment to the RE is accompanied by Sir2 and occurs within the right half that controls chromosome structure, specifically at the promoter of an uncharacterized gene called *RDT1* [27,32]. Deleting a small 100bp region of the *RDT1* promoter is sufficient to eliminate condensin and Sir2 recruitment to the RE, activating *RDT1* expression and disrupting chrIII architecture [27], suggesting the region acts as a locus control region (LCR) that coordinates transcriptional regulation with chromosomal architecture.

*MAT*a-specific enrichment of condensin at the *RDT1* promoter is dependent on a consensus binding site for the MADS-box transcription factor Mcm1 [27]. MADS-box transcription factors generally partner with other proteins to regulate transcription. For example, Mcm1 activates *MAT*a-specific genes in *MAT*a cells. In *MAT*α cells, however, Mcm1 forms a repressive heterodimer with the α2 protein (Mcm1/α2) that inactivates *MAT*a-specific genes [33]. *RDT1* and another non-coding RNA gene (R2) located in the left half of the RE that regulates donor preference are likely controlled by this mechanism [30,34]. Mcm1 also regulates cell cycle genes independent of mating type, including the cyclins *CLN3* and *CLB2* [35]. Since condensin and Sir2 were not enriched at promoters of such genes in previous ChIP-seq datasets [27,36], we hypothesized that additional factors partner with Mcm1 to recruit and load condensin at the *RDT1* promoter. Herein, we unexpectedly find that nucleolar factors cohibin, Fob1, and Tof2, which recruit condensin to the rDNA locus, also directly recruit condensin to the *RDT1* promoter in *MAT*a cells to establish chrIII conformation and augment donor preference.

## Results

**Fig 1:**
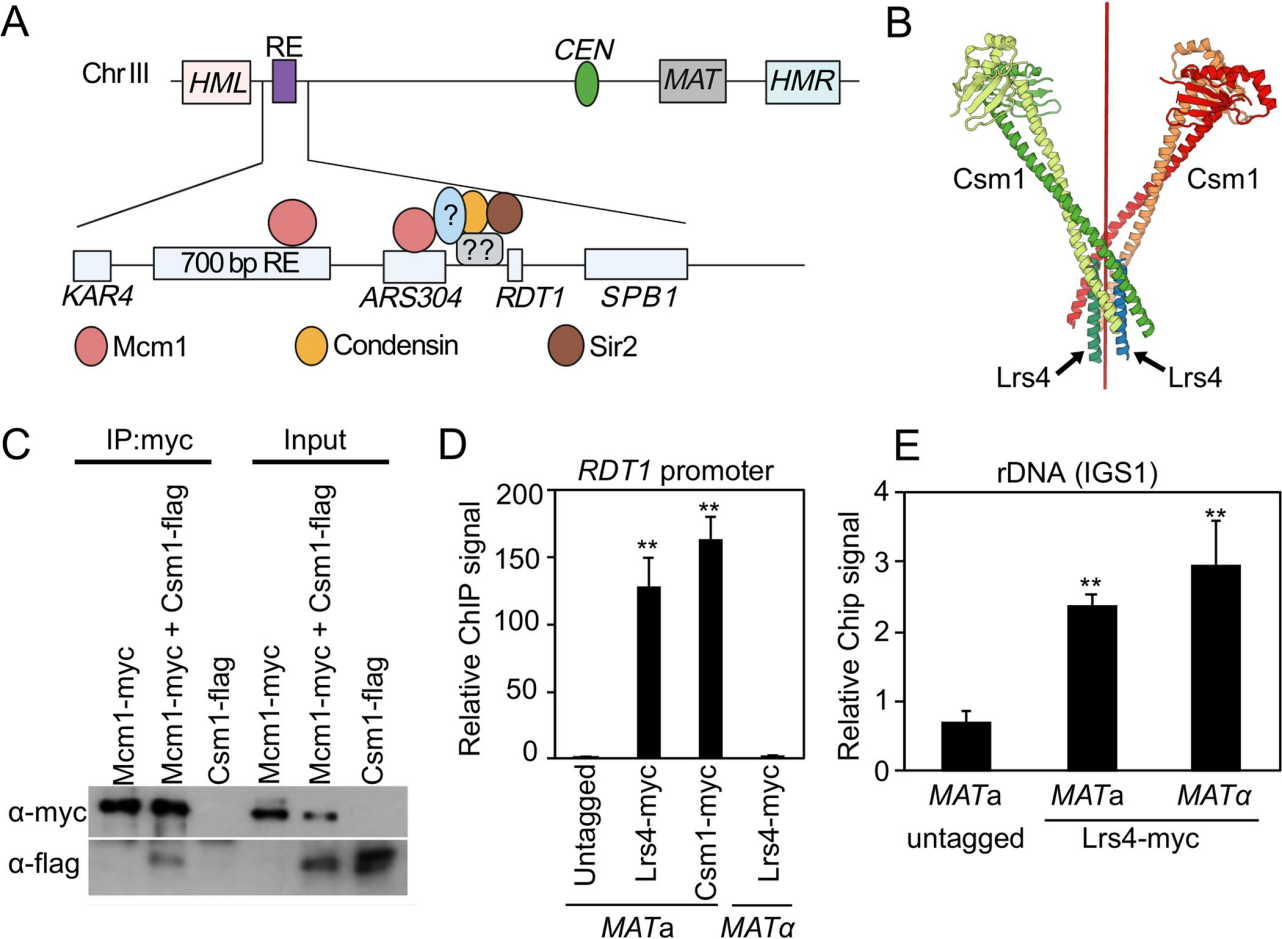
Cohibin binds to the *RDT1* promoter on chrIII specifically in *MAT*a cells. (**A**) Schematic of chrIII in *MAT*a cells. The recombination enhancer (RE) region lies between *KAR4* and *SPB1* and consists of a minimal 700 bp element required for donor preference, two Mcm1/α2 binding sites, *ARS304*, and a Sir2/condensin recruitment site within the *RDT1* promoter. Uncharacterized factors are proposed to assist Sir2 and condensin recruitment by Mcm1. (**B**) X-ray crystallographic structure of cohibin consisting of 4 Csm1 subunits and 2 short Lrs4 subunit peptides, showing extended coiled-coil feature. Red line indicates the axis of symmetry. PDB accession number 3N7N [26]. (**C**) Immunoblot showing that Mcm1-myc coimmunoprecipitates with Csm1-flag. Loaded inputs are 5% of cell lysate used for the IP. (**D-E**) Enrichment of Lrs4-myc and Csm1-myc at the *RDT1* promoter or *IGS1* regions of the rDNA. (Three biological replicates; **p<0.005 relative to untagged strain determined by two-sided t-test assuming unequal variance).

We previously described strong *MAT*a-specific condensin and Sir2 enrichment at the *RDT1* promoter that was dependent on an upstream Mcm1/α2 binding site (*DPS2*; Fig 1A). Deleting 100bp underlying the Sir2 and condensin ChIP-seq peaks (100bpΔ; chrXII coordinates 30701–30800), while leaving the Mcm1/α2 site intact, also significantly disrupted their recruitment [27], suggesting one or more unknown factors cooperate with Mcm1 (Fig 1A). We initially focused on a putative Mcm1-Lrs4 interaction identified from a TAP-tag pulldown screen [37]. Lrs4 and Csm1 are coiled-coil proteins that form an extended v-shaped complex known as cohibin in mitotic cells, or monopolin in meiotic cells (Fig 1B; [24,38]). Lrs4 and Csm1 were of interest because they help recruit condensin and cohesin to the rDNA through interaction with Fob1 [9,19]. Cohibin also physically interacts with Sir2 through the RENT and SIR complexes at the rDNA and telomeres, respectively [19,37]. We confirmed the putative Mcm1-cohibin interaction with co-immunoprecipitations between Myc-tagged Mcm1 and Flag-tagged Csm1 (Fig 1C). Myc-tagged Lrs4 and Csm1 were also highly enriched at the *RDT1* promoter in quantitative ChIP assays, specifically in *MAT*a cells (Fig 1D). As a control, Lrs4-myc enrichment at the rDNA intergenic spacer was not mating-type specific (Fig 1E).

### Cohibin is required for condensin enrichment at the *RDT1* promoter

**Fig 2:**
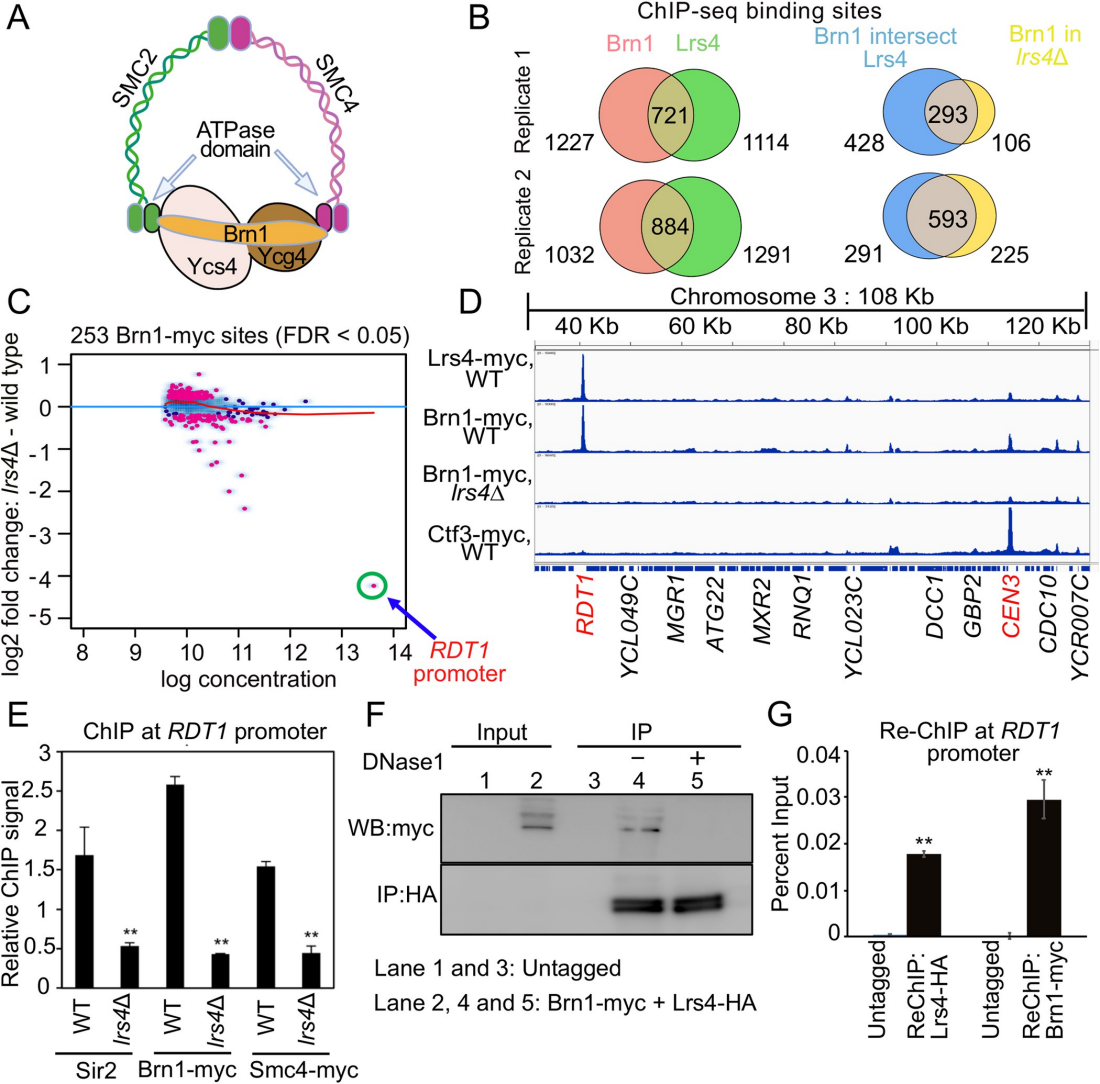
Cohibin recruits condensin to the *RDT1* promoter in *MAT*a cells. (**A**) Schematic of budding yeast condensin subunits. (**B**) Venn diagrams of overlapping ChIP-seq peaks for Brn1-myc and Lrs4-myc in *MAT*a cells from two independent replicates on the left. Among overlapping peaks, the number that change in the *lrs4Δ* mutant are indicated in right Venn diagrams. Peaks that overlap with Ctf3-myc peaks were subtracted from each dataset. (**C**) MA plot indicating differential Brn1-myc binding between WT and *lrs4Δ*. Red dots are peaks significantly different between WT and *lrs4Δ* with FDR < 0.05. Blue dots are peaks not differentially bound. *RDT1* promoter peak is highlighted. (**D**) IGV screenshot of chrIII peaks for Brn1-myc, Lrs4-myc, Ctf3-myc, and Brn1-myc in the *lrs4Δ* mutant. Ctf3 is a centromere-specific control. (**E**) ChIP analysis at *RDT1* showing decreased enrichment of Sir2, Brn1-myc, and Smc4-myc in *lrs4*Δ mutant. (**F**) Co-IP assays from cell lysates were performed with an untagged strain or a strain with Lrs4-HA/Brn1-myc. DNase I digestion disrupts the coimmunoprecipitation. (**G**) Quantitative ChIP-reChIP PCR analysis of condensin (Brn1-myc) and cohibin (Lrs4-HA) co-occupancy at the *RDT1* promoter. N = 3 biological replicates for each ChIP experiment; *p<0.05, **p<0.005 using two-sided t-test assuming unequal variance.

The interaction of cohibin with Mcm1 and its enrichment at *RDT1* in *MAT*a cells suggested a model whereby the extended coiled-coil structure of cohibin (Fig 1B) acts as a bridge between Mcm1 and the condensin/Sir2 complexes (Fig 1A, blue oval). To investigate if cohibin was contributing to condensin recruitment specifically at *RDT1*, or more generally across the genome, we performed ChIP-seq for Myc-tagged subunits of condensin (Brn1-13xmyc; Fig 2A) or cohibin (Lrs4-13xmyc) in *MAT*a cells. Myc-tagged centromeric protein, Ctf3, was used as a control for “hyper-ChIPable” sites that appear in yeast ChIP-seq datasets [39]. We identified >700 sites that overlapped between Lrs4-myc and Brn1-myc after excluding Ctf3-myc peaks (Fig 2B, left Venn diagrams). A large proportion of the overlapping peaks showed differential Brn1-myc binding in the absence of Lrs4 (Fig 2B, right Venn diagrams). If cohibin was recruiting condensin genome-wide, then *lrs4Δ* should reduce Brn1-myc enrichment at most sites. However, only 7 Brn1-myc sites had a log2 fold change below -1 (Fig 2C), suggesting that cohibin-dependent recruitment of condensin is locus-specific. The differential Brn1-myc sites are listed in S1 Table.

Interestingly, the strongest Lrs4-dependent binding site was the *RDT1* promoter (Fig 2C). Overlapping distinctive peaks for Lrs4-myc and Brn1-myc were clearly observed at *RDT1* on chrIII, with smaller overlapping peaks at several other loci (Fig 2D). As predicted from the MA plot in Fig 2C, the strong Brn1-myc peak at *RDT1* was eliminated in the *lrs4Δ* mutant (Fig 2D). A similar ChIP-seq pattern was observed for the rDNA at IGS1 (S1A Fig), where cohibin was known to recruit condensin [9]. We validated condensin loss at *RDT1* by quantitative ChIP-qPCR for Brn1-myc and Smc4-myc subunits in the *lrs4Δ* mutant (Fig 2E) and observed similar depletion for endogenous Sir2 (Fig 2E). Enrichment of Lrs4 at the *RDT1* promoter was highly specific, as confirmed by a lack of detectable Lrs4-myc ChIP signal at the adjacent *RDT1* open reading frame (S1B Fig).

The Ctf3-myc control was not enriched at *RDT1*, but as expected, was strongly bound to the chrIII centromere (Fig 2D; *CEN3*). Ctf3-myc also produced non-specific peaks that overlapped with Lrs4-myc and Brn1-myc (Fig 2D), likely examples of “hyper-ChIPable” loci. Cohibin co-localizes with condensin at mitotic and meiotic kinetochores in *S*. *cerevisiae* and *S*. *pombe* where they promote proper chromosome segregation [40,41]. However, only in *S*. *pombe* was cohibin (Pcs1/Mde4) directly implicated in recruiting condensin to kinetochores [42]. Here, we observed Lrs4-myc and Brn1-myc enrichment at most centromeres, including *CEN3* (Fig 2D). Moreover, the Brn1-myc peak at *CEN3* was clearly diminished in the *lrs4Δ* mutant (Fig 2D), suggesting that cohibin may also participate in condensin recruitment to centromeres in *S*. *cerevisiae*, though these sites were discarded from Fig 2B and 2C because they are also Ctf3 binding sites. To confirm that cohibin and condensin co-associate on chromatin, as predicted for a recruitment/loading function, we first performed a co-immunoprecipitation experiment showing that the interaction between Brn1-13xmyc and Lrs4-3xHA was eliminated by Dnase I treatment (Fig 2F). Second, a ChIP-reChIP assay demonstrated their co-occupancy on the *RDT1* promoter (Fig 2G).

### Fob1 recruits cohibin and condensin to chromosome III

**Fig 3:**
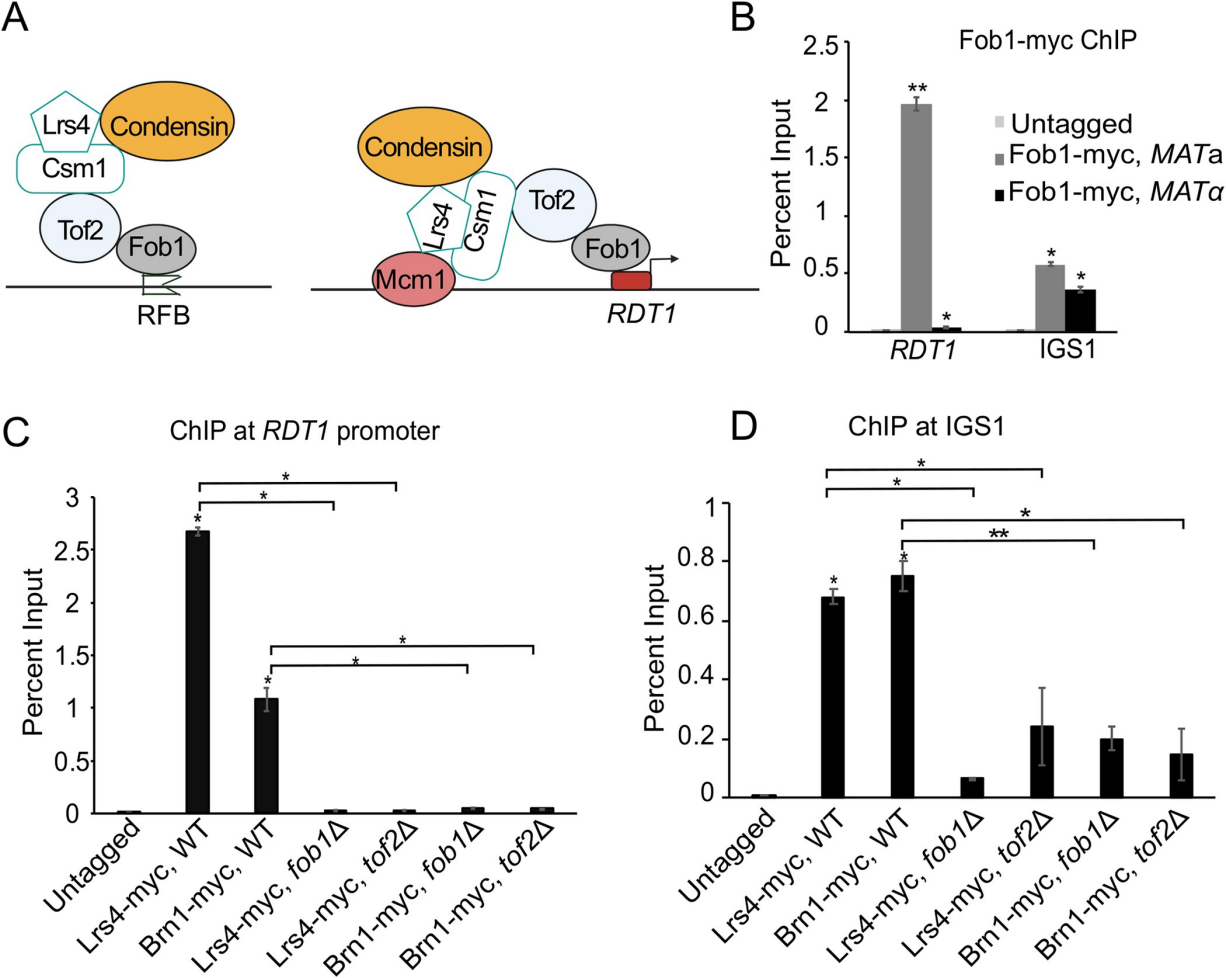
Fob1 recruits cohibin and condensin to the *RDT1* promoter. (**A**) Hierarchical models of condensin recruitment to the rDNA and *RDT1* promoter. (**B**) ChIP of Fob1-myc at the *RDT1* promoter and *IGS1* in *MAT*a and *MAT*α cells. (**C-D**) ChIP analysis of Lrs4-myc and Brn1-myc enrichment at the *RDT1* promoter or *IGS1* showing dependence on *FOB1* and *TOF2*. N = 3 biological replicates for each ChIP experiment; *p<0.05, **p<0.005 using two-sided t-test assuming unequal variance.

If interaction between Mcm1 and cohibin was the sole mechanism of bringing condensin to *RDT1*, then Lrs4-myc should be enriched at other known Mcm1 sites, including an Mcm1 site (DPS1) within the left half of the RE. This was not the case (Fig 2D). Instead, our model for condensin recruitment to *RDT1* allowed for contributions from one or more additional factors, including other site-specific DNA binding proteins (Fig 1A, gray rounded rectangle). Condensin and cohibin are both targeted to the RFB site in IGS1 by hierarchical interactions with Fob1 and Tof2, with Fob1 providing the site-specific DNA binding activity (Fig 3A, left diagram; [9,43]). We therefore hypothesized that a similar hierarchy of Fob1-dependent recruitment occurred at *RDT1*, with mating-type specificity added by Mcm1 (Fig 3A, right diagram). Fob1 had no previously known function outside the nucleolus/rDNA, but in ChIP assays we observed exceptionally strong *MAT*a-specific enrichment of Fob1-13xmyc at *RDT1*, compared to mating-type independent enrichment at IGS1 (Fig 3B). ChIP was also used to test whether a similar hierarchy of Fob1-dependent condensin recruitment was occurring at *RDT1* (Fig 3C) and rDNA (Fig 3D). Deleting *FOB1* or *TOF2* eliminated Lrs4-myc and Brn1-myc enrichment at *RDT1*, and a similar pattern was observed at the rDNA (IGS1), where the Lrs4-myc and Brn1-myc depletion was not absolute, perhaps because rDNA ChIP assays average the PCR signal from ~150 repeats, or additional factors are involved in condensin and cohibin recruitment to the rDNA.

### Fob1 directly binds to *RDT1* promoter sequence

**Fig 4:**
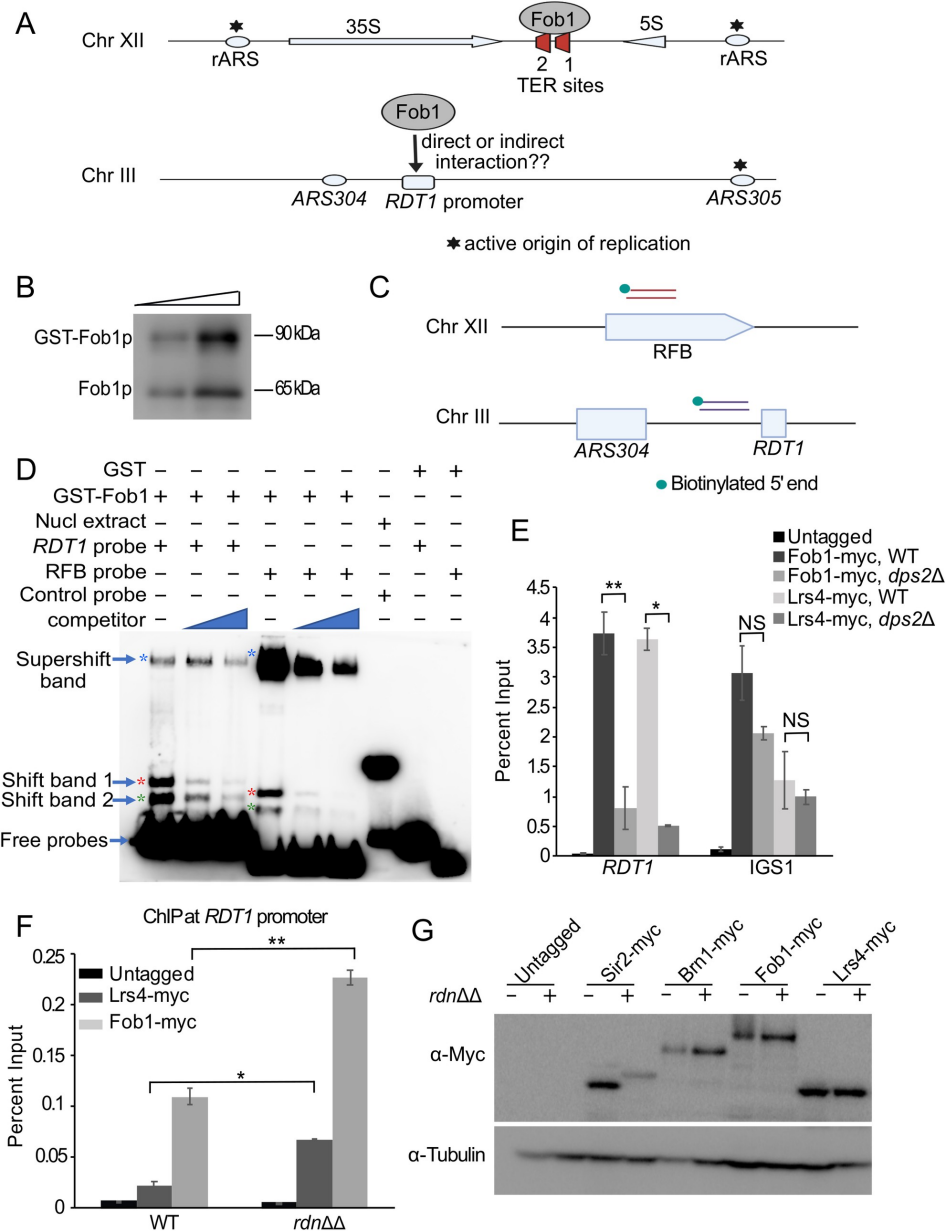
Recombinant Fob1 directly binds to the *RDT1* promoter sequence *in vitro*. (**A**) Schematic of Fob1 binding to TER1 and TER2 sites of the replication fork block (RFB), and the *RDT1* promoter. (**B**) Western blot of yeast-purified GST-Fob1p using α-Fob1 antibody. (**C**) Schematic for the design of biotin labelled probes used for EMSA. (**D**) Representative EMSA with biotin-labeled probes and purified GST-Fob1p, GST, or nuclear extract positive control. Competitor probes were unlabeled. The *RDT1* probe is 57 bp and the RFB probe is 42 bp. Positive control for gel shift was EBNA nuclear extract and EBNA DNA probe from the gel-shift kit. An additional replicate and quantitation are shown in S2 Fig. (**E**) ChIP assay showing Fob1-myc or Lrs4-myc enrichment at the *RDT1* promoter or the RFB in IGS1 when the adjacent Mcm1 binding site was deleted (*dps2Δ*). (**F**) ChIP of Lrs4-myc and Fob1-myc at the *RDT1* promoter in a strain (NOY891) lacking the rDNA array (*rdnΔΔ*) or W303 WT control. ChIP signal is relative to the input chromatin signal expressed as percent input. (**G**) Western blot showing steady state Brn1-myc, Lrs4-myc, Fob1-myc, and Sir2-myc levels in WT and *rdnΔΔ* strains. The small size difference for Sir2-myc protein between WT and *rdnΔΔ* strains was due to one less myc repeat in the WT. α-tubulin is used as a loading control. N = 3 biological replicates for each ChIP experiment; *p<0.05, **p<0.005 using two-sided t-test assuming unequal variance.

Fob1 binding to rDNA occurs at the RFB site in IGS1, consisting of 2 replication fork termination sites (Fig 4A; TER1 and TER2) [44]. These sites do not match sequence within the *RDT1* promoter, and due to TER1 and TER2 being the only previously known Fob1 binding sites, there is no consensus binding sequence for this protein. To test if Fob1 directly binds the *RDT1* promoter (Fig 4A, lower), we performed electrophoretic mobility shift assays (EMSA). GST-Fob1 was overexpressed in yeast and purified by affinity chromatography (Fig 4B), then incubated *in vitro* with small biotin-labeled PCR products derived from the *RDT1* promoter (chrIII coordinates 30712 to 30768) or RFB (chrXII coordinates 460533 to 460574) (Fig 4C). Purified GST-Fob1, but not GST alone, produced two closely spaced lower band shifts (1 and 2) as well as a higher supershifted band with both probes (Fig 4D). The GST-Fob1 preparation contained both GST-Fob1 and free Fob1 (Fig 4B), so bands 1 and 2 likely represent binding by both species, each of which was competed away by the respective unlabeled probes (Figs 4D and S2). The supershifted band was the predominant RFB product and showed significant competition from unlabeled probe (Figs 4D and S2), whereas the *RDT1* supershifted band was a minor product and poorly competed. Since the RFB probe contains two specific Fob1 binding sites (TER1 and TER2), the supershift likely represents specific occupation of both sites or multimerization, consistent with Fob1 dimerization mediating ‘chromosome kissing’ between pairs of TER sites [15]. Specific Fob1 binding to *RDT1* may instead be limited to a single site (bands 1 and 2) with the supershift representing additional non-specific binding to the probe. In summary, these results show that Fob1p directly binds to the *RDT1* promoter sequence, consistent with the hierarchical model for condensin recruitment defined by ChIP (Fig 3A).

Since Fob1 bound directly to the *RDT1* probe *in vitro*, this raised the question of why its enrichment at *RDT1 in vivo* was specific to *MAT*a cells. We hypothesized that Mcm1 was providing the *MAT*a specificity by acting as a pioneer transcription factor to open local chromatin structure and allow Fob1 access to its binding site. To test this idea, we deleted the Mcm1 binding site to prevent Mcm1 recruitment [27], and tested for Fob1-myc or Lrs4-myc binding by ChIP. As shown in Fig 4E, Fob1-myc and Lrs4-myc recruitment to *RDT1* was significantly reduced, but there was no effect at IGS1 in the rDNA. This result placed Mcm1 at the base of the hierarchical condensin recruitment at *RDT1*, again consistent with the model in Fig 3A.

We next tested if *RDT1* was in competition with repetitive rDNA for the predominantly nucleolar condensin recruitment complex, especially since there appeared to be stronger affinity for the RFB EMSA probe (supershifted band in Fig 4D). Fob1 and Lrs4 were C-terminally tagged with 13xMyc in a strain lacking the chromosomal rDNA array (*rdnΔΔ*) and kept viable by a plasmid expressing 35S rRNA precursor from a galactose-inducible RNA Pol II promoter [45]. ChIP assays for *RDT1* promoter enrichment in the WT and *rdnΔΔ* strains were then performed (Fig 4F). Fob1-myc and Lrs4-myc showed significantly improved enrichment at *RDT1* in the *rdnΔΔ* mutant while the overall fusion protein levels were unaffected (Fig 4F and 4G). This contrasted with a Sir2-myc control, which showed reduced protein in the *rdnΔΔ* mutant, as predicted for strains with low rDNA copy number [46,47]. From this experiment we concluded that *RDT1* competes with the rDNA for limiting Fob1 and cohibin, implying that rDNA copy number could potentially regulate chromosome III structure and mating-type switching.

### Cohibin and Fob1 augment donor preference

**Fig 5:**
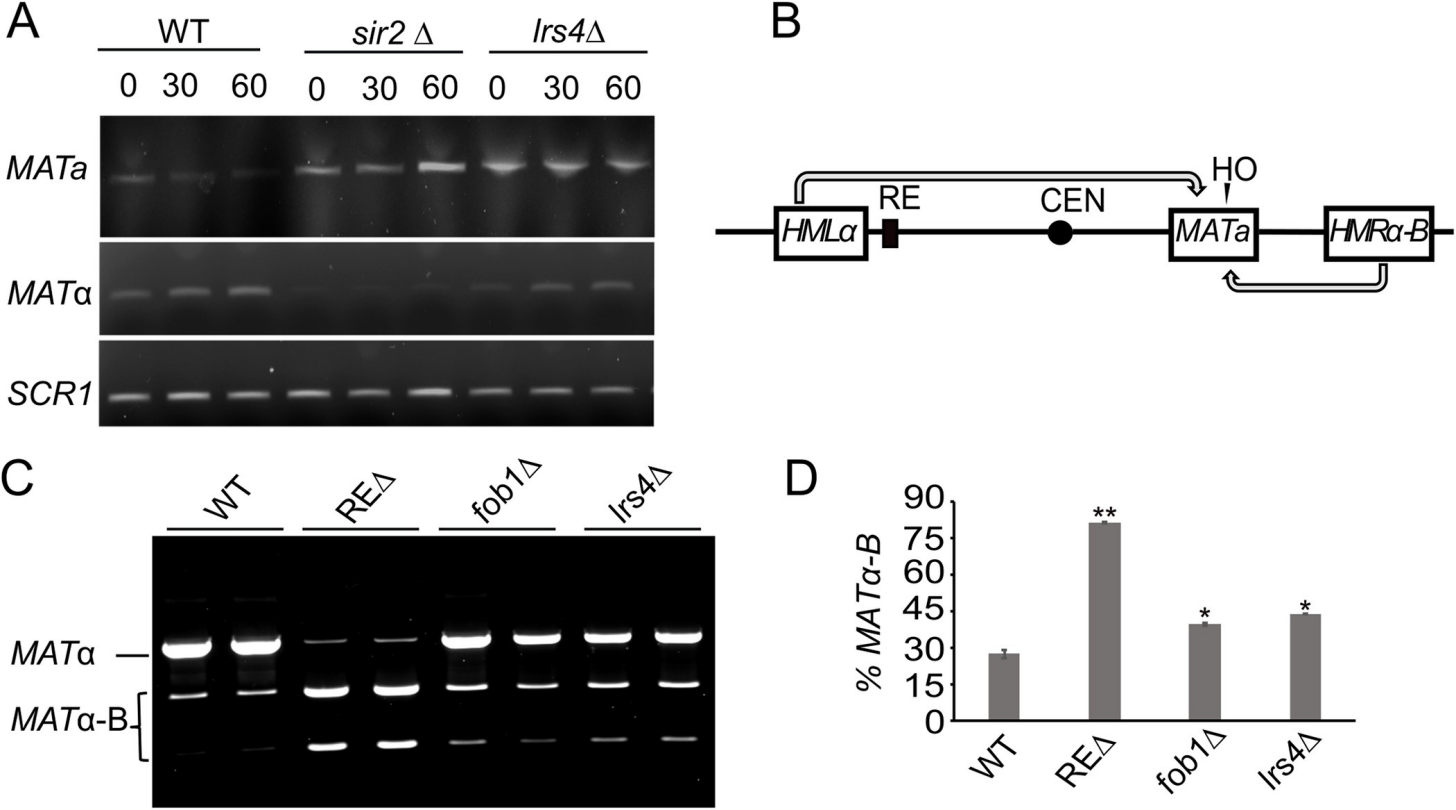
Contributions of Lrs4 and Fob1 to mating-type switching. (**A**) Time-course of mating-type switching in WT (ML447), *sir2*Δ (ML458) and *lrs4*Δ (MD69) showing PCR detection of *MAT*a and *MAT*α. *SCR1* is used as a control for input genomic DNA. (**B**) Schematic of the donor preference assay that distinguishes usage of *HML*α or an engineered *HMR*α-B cassette as the donor template by digestion of a *MAT*α PCR product with *Bam*HI. (**C**) Donor preference assay with WT (XW652), *lrs4Δ* (SY762), *fob1Δ* (MD135), or *REΔ* (XW676) showing *Bam*HI digestion of the *MAT*α PCR product after switching. Upper band indicates normal donor preference switching to *MAT*α using *HML*α as the template. Digested bands indicate improper switching to *MAT*α-B using *HMR*α-B as the template. (**D**) The percentage of *MAT*α-B PCR product is quantified in the bar graph. Significant increases compared to WT = *p<0.05, **p<0.005 (n = 3), as calculated by 2-tailed student t-test.

Acute depletion of Brn1 fused to an auxin-inducible degron (AID) slows the rate of mating-type switching and moderately impairs donor preference [27]. Since cohibin and Fob1 were required for condensin recruitment at chrIII (Figs 2E and 3C, respectively), we predicted a similar effect on switching would occur for *lrs4Δ* or *fob1Δ* mutants. As shown in Fig 5A, conversion from *MAT*a to *MAT*α was indeed slowed in an *lrs4Δ* mutant, but less severely than *sir2Δ*, which strongly inhibits switching due to HO endonuclease cutting at the *HML* and *HMR* donor templates. We also tested the impact of *lrs4Δ* or *fob1Δ* on donor preference using an assay that distinguishes between *HML*α and *HMR*α-B (marked with *Bam*HI site) donors by digesting *MAT*α PCR products with *Bam*HI ([48]; Fig 5B). As predicted, the *lrs4Δ* and *fob1Δ* mutants weakened donor preference (*Bam*HI digestion of *MAT*α PCR product), not severely like the REΔ control strain (Fig 5C and 5D), but comparable to the switching phenotypes previously observed with condensin depletion [27]. Condensin recruitment by Fob1 and cohibin therefore appears to augment donor preference activity, which is primarily directed by the left half of the RE [29].

### Cohibin and Fob1 dictate the conformation of chromosomes III and XII

**Fig 6:**
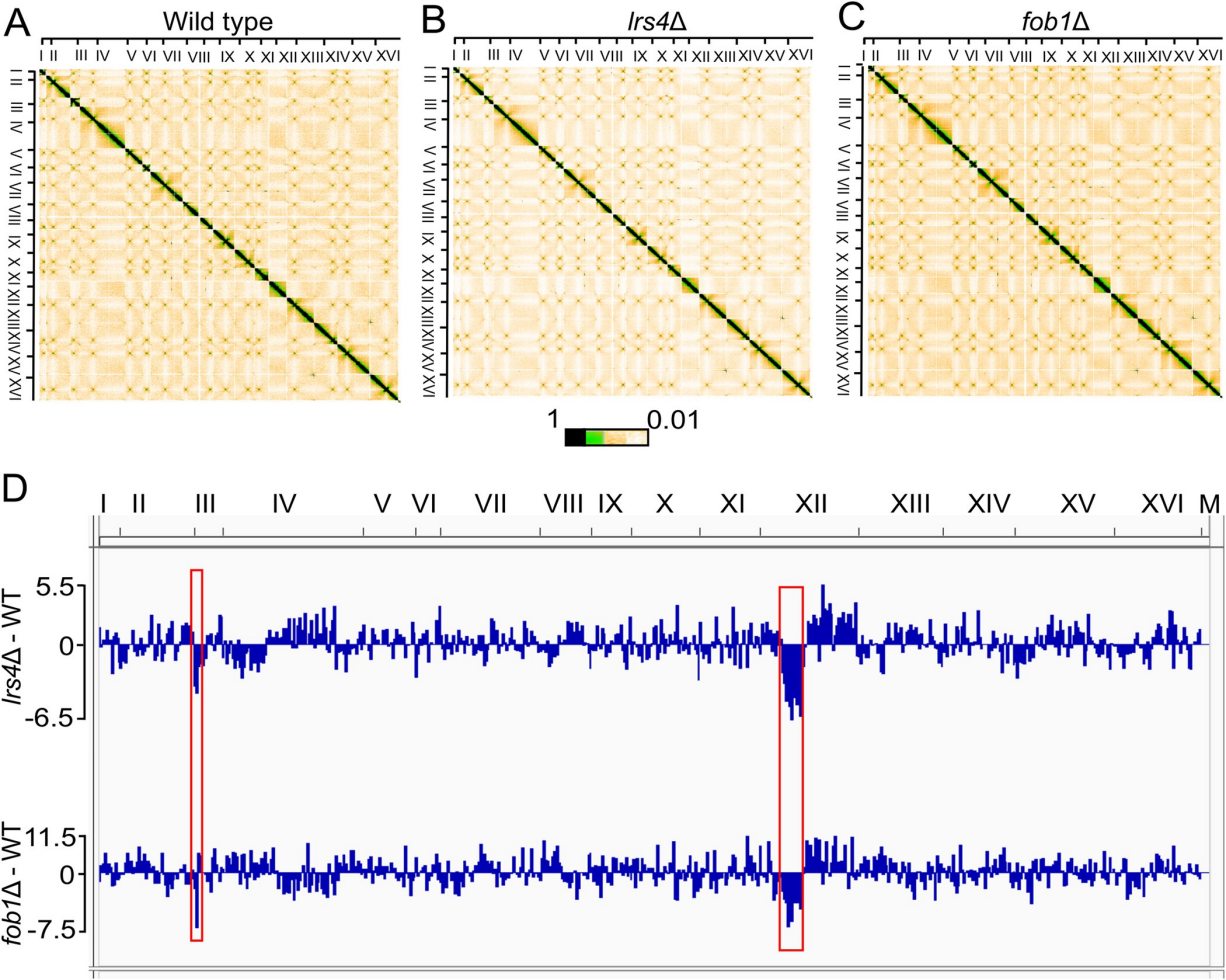
Micro-C XL analysis of the yeast genome in *lrs4Δ* and *fob1Δ* mutants. (**A-C**) Genome-wide interaction heatmaps of Micro-C XL data in exponentially growing *MAT*a wild type, *lrs4*Δ and *fob1*Δ cells, respectively. Each pixel represents normalized chromatin contacts between 10 kb bins. The color scale indicates maximum and minimum interaction counts from 10kb intervals across the genome. (**D**) Differential Chromatin Interaction (DCI) scores were calculated for normalized contact frequencies of 5kb target across +/-200kb flanking regions, comparing *lrs4Δ* or *fob1Δ* mutants with WT. Sequencing reads were pooled from 3 WT and 3 *lrs4Δ* biological replicates, and 2 *fob1Δ* replicates. The negative DCI scores observed in chromosome III and chromosome XII are boxed in red.

Given the requirements for cohibin and Fob1 in condensin recruitment to the *RDT1* promoter on chrIII and the rDNA on chrXII, and their similar contributions to donor preference, we hypothesized that deleting *LRS4* or *FOB1* should significantly disrupt the structure of both chromosomes. Genome-wide chromatin contacts were analyzed by Micro-C XL [49]. Normalized interaction frequencies within 10 kb bins were first visualized in heatmap plots for WT, *lrs4Δ* and *fob1Δ* strains. Interchromosomal interaction patterns were generally similar between the three strains (Fig 6A–6C). For example, absence of Lrs4 or Fob1 did not reduce the number of centromere-centromere or telomere-telomere interactions among the top 10% of overall interactions (S3A and S3B Fig), indicating gross interchromosomal organization remained intact, at least in the context of the asynchronously grown cells used in this experiment. It remains possible that interchromosomal contacts could be different at certain points of the cell cycle.

We also quantified differential intrachromosomal contacts between WT and the *lrs4Δ* or *fob1Δ* mutants using BART3D [50]. A differential chromatin interaction (DCI) score was calculated for each 5 kb segment across the genome to measure the difference in normalized contact between each segment and its +/-200 kb flanking regions (Fig 6D). Segments with the strongest negative DCI scores, representing the most decreased interactions in the *lrs4Δ* compared to WT (S3C Fig), were predominantly from a region of chrXII between the centromere and rDNA that forms a condensin-dependent TAD [51–53]. The *RDT1*-containing chrIII segment (coordinates 30000 to 35000) was also among the 20 most negative DCI scores in the *lrs4Δ* mutant (Figs 6D and S3E). The same regions of chrIII and XII were also among the most negatively affected in the *fob1Δ* mutant (Fig 6D), including the RE-containing chrIII segment as the top hit (S3D and S3F Fig). Taken together, we conclude that Lrs4 and Fob1 both dictate chrIII and XII organization, most likely through localized recruitment and loading of condensin.

**Fig 7:**
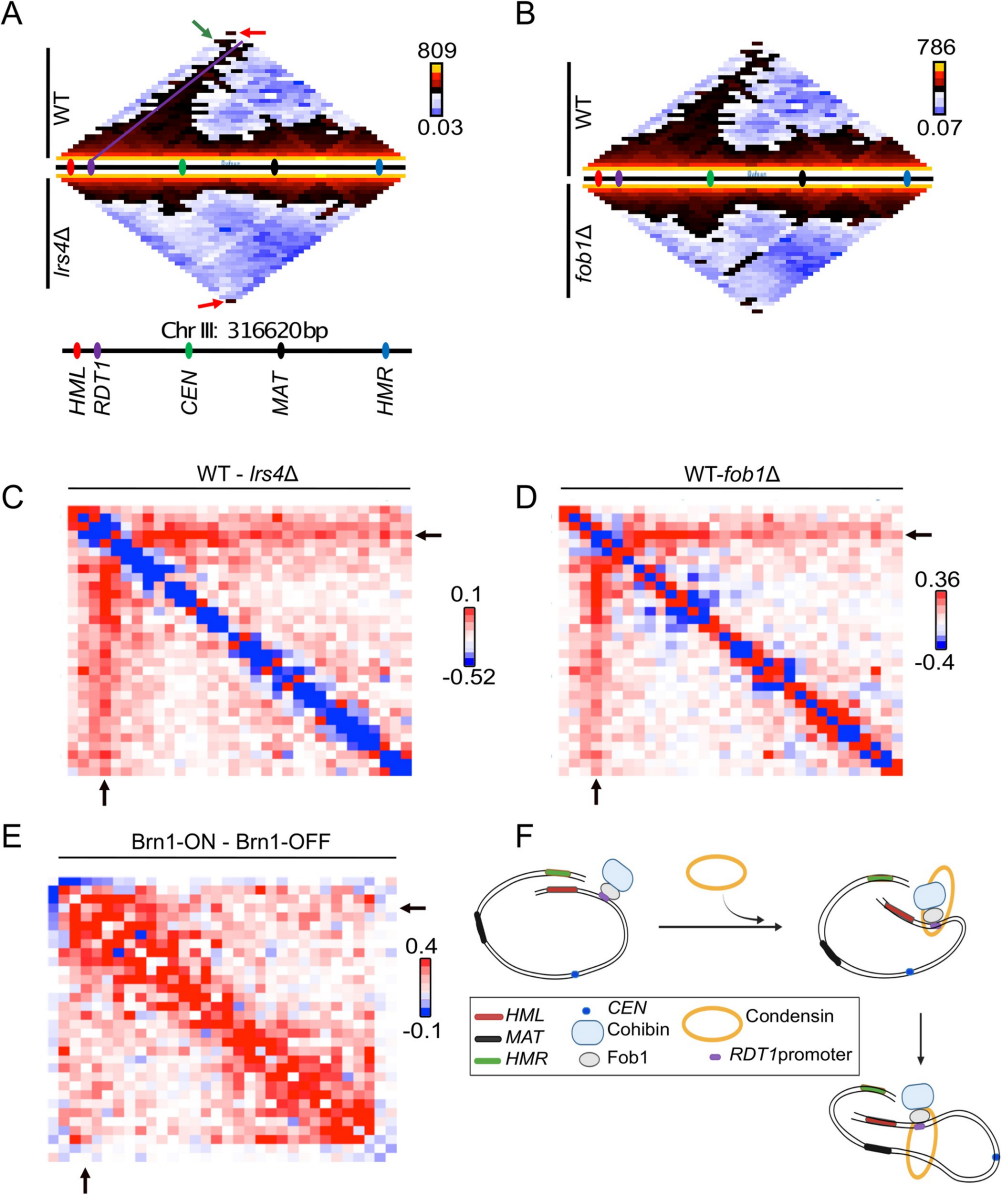
Lrs4, Fob1, and condensin control chromosome III conformation in *MAT*a cells. (**A**) Micro-C XL interaction heat map (iteratively corrected) for chromosome III at 10 kb resolution comparing WT (top) and *lrs4Δ* (bottom) strains. In the WT map, a green arrow indicates the *HML-HMR* interaction, a purple line indicates interaction of the *RDT1*-containing bin with the rest of chrIII. Red arrows indicate the telomere-telomere interaction. (**B**) ChrIII interaction heat maps for WT and *fob1Δ* strains. The color scales in panels A and B indicate maximum and minimum interaction counts from 10kb intervals across the genome. (**C**-**D**) Subtraction of *lrs4Δ* or *fob1Δ* interaction frequencies from WT reveals a rightward diminishing gradient of interaction in the WT strain anchored at the *RDT1* bin. Red shading indicates high in WT and blue shading indicates higher in the mutant. (**E**) Subtraction of the Brn1 anchor-away interaction frequencies from non-depleted control. Black arrows indicate the *RDT1* bin. Subtraction values in panels C-E are arbitrarily scaled to be in the linear color scale range. Sequencing reads were pooled from 2 or 3 biological replicates. (**F**) Loop extrusion model for condensin anchored to the *RDT1* promoter by Fob1 and cohibin, bringing *HML* in proximity with *MAT*a.

Previous 3C and Hi-C analyses of chrIII conformation in *MAT*a and *MAT*α cells identified a prominent interaction between the subtelomeric *HML* and *HMR* loci [31,54]. Deleting the *RDT1* promoter (100bpΔ mutant) was sufficient to disrupt the *HML*-*HMR* interaction at least partially in *MAT*a cells, freeing *HMR* to interact with the *MAT*a locus [27]. This mutant also disrupted contacts between *HML* and the right arm of chrIII, including *MAT*a [27]. To test if these contacts were also disrupted by *lrs4Δ* or *fob1Δ*, an iteratively corrected chrIII Micro-C XL contact map (10kb bins) for each mutant was directly compared to WT (Fig 7A and 7B). Sequencing reads from at least 2 biological replicates were pooled to maximize resolution. Combined with the higher resolution of Micro-C XL, the WT pattern now delineated an apparently rightward-extended path of interaction anchored at the RE-containing bin, as well as the expected *HML*-*HMR* (green arrow) and telomere-telomere (red arrows) interacting bins, which were offset from the line of RE-anchored interactions. Importantly, the *HML*-*HMR* and RE-anchored interactions were disrupted in the *lrs4Δ* and *fob1Δ* mutants, but the telomere-telomere interaction was retained (Fig 7A and 7B, red arrows). Subtraction plots of the pooled Micro-C XL data (WT minus mutant) revealed a gradient along the RE-anchored interaction path (Fig 7C and 7D), consistent with Lrs4- or Fob1-recruited condensin mediating loop extrusion in a rightward direction. Individual replicate iterative correction interaction maps and subtraction plots are provided in S4 Fig. Loop extrusion by the yeast condensin complex proceeds unidirectionally *in vitro* [2], but given the proximity of *RDT1* with the left telomere (~30kb), we cannot formally rule out two-sided loop extrusion *in vivo*. To confirm that condensin loss directly alters chrIII structure, we re-analyzed 3C-seq data obtained from asynchronous cultures where Brn1 was depleted from the nucleus using anchor-away [53]. As shown in Fig 7E, Brn1 depletion more generally disrupted chrIII organization as compared to *lrs4Δ* and *fob1Δ*, though contacts between the left and right arms were also disrupted, consistent with loss from the *RDT1* region.

Since Fob1 and cohibin recruit condensin to the rDNA (S5A Fig schematic), and a condensin-dependent TAD is formed on chrXII between the rDNA and centromere [52,53], we predicted that deleting *LRS4* or *FOB1* would disrupt this TAD. Micro-C XL interaction subtraction plots were generated for chrXII, indeed revealing defects in TAD formation for the *lrs4Δ* and *fob1Δ* mutants (S5B and S5C Fig), similar to the defect observed following Brn1 depletion by anchor-away (S5D Fig; [53]). All three mutant conditions also resulted in more frequent interactions between the disrupted TAD region and the right side of chrXII (S5D Fig; [53]). Lastly, TAD disruption in the *fob1Δ* mutant coincided with substantial self-interaction within the right side of chrXII, perhaps due to redistribution of the released cohibin, condensin, cohesin, and potentially other unknown factors. Taken together, we conclude that similar to the rDNA locus, Fob1 and cohibin recruits and anchors condensin to the *RDT1* promoter where it folds chrIII into a specialized conformation through loop extrusion (Fig 7F).

## Discussion

Interphase chromosomes of multicellular organisms are organized into 3-dimensional domains ranging in size from kilobase scale chromatin loops to megabase scale topologically associated domains (TADs). Yeast chromosomes are also 3-dimensionally organized, but at a much smaller scale. Computational modeling, imaging, and Hi-C analyses predict a Rabl-like conformation whereby centromeres are clustered with the duplicated spindle pole bodies, and telomeres clustered into several perinuclear foci [55]. Tethered centromeres limit spatial mobility of the chromosome arms [56], which essentially fill limited space within the small yeast nucleus, resulting in chromosome “territories” that can be largely explained by geometrical constraints [57]. Despite such limitations, there are still multiple biochemically generated architectural features within the yeast genome, including abundant self-associating chromatin domains detected by Micro-C that only span across one to five genes and are dependent on chromatin remodeling factors [49,58]. There are also biochemically generated large-scale features, such as the rDNA array and the condensin-dependent TAD formed between the centromere and rDNA on chrXII (S5 Fig; [51–53,55]). The *MAT*a- and *MAT*α-specific conformations of chrIII comprise the other major large-scale architectural features of the budding yeast genome [31,54].

### A shared mechanism of condensin recruitment between the rDNA and chrIII

Lrs4 (Loss of rDNA silencing 4) was first identified through a screen for genes that function in Sir2-dependent rDNA silencing [59]. It was later found to be a subunit of the meiotic monopolin complex (Lrs4/Csm1/Mam1) responsible for monopolar attachment of microtubules to kinetochores during meiosis I [25]. We became interested in cohibin (Lrs4/Csm1) as a possible condensin and Sir2 recruitment factor for chrIII in *MAT*a cells because pull-down experiments with TAP-tagged Lrs4 and Csm1 subunits identified Mcm1 as a putative interacting protein [37]. Additionally, Lrs4, Csm1, and Tof2 were previously identified in a protein interaction network with Fob1 and the RENT complex [19], and to be involved in both cohesin and condensin recruitment at IGS1 in the rDNA [9,19]. While primarily localized at the rDNA/nucleolus, some of these factors have known functions outside the nucleolus. The Cdc14 subunit of RENT is partially redistributed outside the nucleolus during early anaphase by the FEAR network [60], and then further released at late anaphase by the Mitotic Exit Network (MEN) [61]. Similarly, cohibin is released from the nucleolus by the MEN network during anaphase, relocalizing to kinetochores to ensure faithful chromosome segregation [40]. Cohibin also interacts with telomeres where it promotes their clustering at the nuclear periphery during S and G1 through association with inner nuclear membrane proteins [37]. The RE/*RDT1* promoter represents a new functional cohibin target. It remains to be determined if this locus plays any role in the FEAR or MEN networks.

### Fob1 function at the RE

Recruitment of cohibin and condensin to the RE is the first non-nucleolar function described for Fob1. Cohibin recruitment to telomeres is dependent on the SIR proteins, not Fob1, and is enhanced in a *fob1Δ* mutant due to release of Sir2 from the rDNA [37]. There is also no evidence of cohibin recruiting condensin to telomeres, and we have not detected significant condensin enrichment at telomeric regions in ChIP-seq datasets. Therefore, the finding that Fob1 is required for cohibin and condensin recruitment at *RDT1* makes the underlying mechanism most similar to recruitment of these factors at rDNA. In such a model, Fob1 provides the sequence-specific DNA binding function, either at the *RDT1* promoter or RFB TER sites at the rDNA. A key distinction is the Mcm1/α2 binding site upstream of *RDT1* controls the mating-type specificity. We propose that formation of an Mcm1/α2 heterodimer at the *RDT1* promoter in *MAT*α cells forms repressive chromatin structure that prevents DNA binding by Fob1, blocks cohibin from interacting with Fob1 and Mcm1, or perhaps blocks a putative Mcm1-Fob1 association. An inability to load condensin therefore prevents formation of the chrIII structural features observed in *MAT*a cells. The absence of Mcm1/α2 regulation at the rDNA makes cohibin, condensin, and RENT recruitment independent of mating-type.

At the rDNA, Fob1 also acts as a unidirectional DNA replication fork block factor. Fob1 bound to the TER1/TER2 sites blocks forks moving in opposite direction of RNA Pol I transcription [10,11]. An autonomously replicating sequence (*ARS304*) is located immediately upstream of the Fob1 binding site at *RDT1* and is marked by the Mcm1/α2 binding site. Although bound by Mcm1, ARS304 and all other ARS elements to the left of *RDT1* are inactive [62], implying the region is replicated from the highly active *ARS305* located to the right of the RE. The RE or *RDT1* promoter region has not been implicated in fork blocking in WT strains, though pausing has been detected in a strain lacking Rrm3, a helicase that normally facilitates replication through non-histone protein complexes [63]. Fork block activity of Fob1 at the rDNA is genetically separable from its role in silencing via RENT recruitment [64], so it is possible that both activities occur at *RDT1* under specific conditions such as a particular cell cycle stage or mating-type switching.

Fob1 and cohibin could potentially function in mediating chromatin organization of chrIII separate from their roles in recruiting condensin. For example, Fob1 was previously shown to mediate “chromosome kissing” or looping between rDNA repeats through inter-repeat Fob1-Fob1 protein interactions [15]. Cohibin is also thought to link different chromatin regions through protein-protein interactions mediated by globular domains of the Csm1 subunit, a mechanism by which cohibin-bound telomeres are clustered at the nuclear periphery through interaction with inner nuclear membrane proteins [24]. Given that *RDT1* is located only 16 kb from *HML*, it is possible that the cohibin recruited to *RDT1* by Fob1 could potentially contribute similarly to the known localization of *HML* near the nuclear periphery [65]. There is also evidence for the RE (left half) modifying the stability of gene silencing at *HML* [66], suggesting cross-talk between the two loci.

### Fob1 and cohibin organize chromosome XII structure

Brn1 depletion disrupts the TAD-like domain between the centromere of chrXII and the rDNA (S5D Fig; [53]). Unfortunately, the repetitive rDNA is not visible in Hi-C and related experiments, but we can speculate that this region of chrXII closely associates with the rDNA array, especially since it forms during anaphase when the rDNA is also condensed [52]. Evidence for rDNA association with the chrXII centromere and the TAD region was obtained by *in silico* 4C analysis that considered the rDNA repeats as a single locus, revealing that the rDNA associated more frequently with the left side of chrXII than the right side, especially with the TAD region [53]. This directionality could be related to the direction of rDNA transcription by RNA polymerase I, which progresses toward the centromere side of chrXII, potentially generating positive supercoiling downstream. Consistent with such a directional model, we previously observed strong chromatin differences in the form of Sir2-dependent transcriptional silencing between the left and right rDNA-flanking chrXII regions that were driven by the direction of RNA Pol I transcription [67]. Silent chromatin had spread out of the rDNA and into the left flanking unique sequence, but not into the right flank [67]. In yeast, the nuclear envelope does not break down during mitosis and rDNA transcription by Pol I continues even though the chromatin is more condensed. Perhaps anaphase specificity of the *CEN12*-rDNA TAD is meant to isolate such an unusual structure from the rest of the genome during mitosis, as evidenced by contacts with the right side of chrXII when Fob1, Lrs4, or Brn1 are deleted or depleted (S5B–S5D Fig). Further evidence that the TAD structure is associated with the rDNA comes from the fact that deleting *FOB1* or *LRS4*, which prevents condensin recruitment to the rDNA, also significantly weakens the TAD structure, especially close to the rDNA (S5 Fig).

### A model for condensin-mediated loop extrusion associated with mating-type switching

SMC complexes such as condensin and cohesin generate chromatin loops through ATP-dependent loop extrusion. Results herein predict that condensin anchored at the *RDT1* promoter performs loop extrusion in *MAT*a cells that extends toward the centromere and *MAT*a locus on chrIII (Fig 7F). While we could not perform a time course of increased DNA juxtaposition from the condensin-bound anchor point in the RE, as was previously done for the *Bacillus subtilis* SMC (condensin) complex loaded onto a specific ParB recruitment site on the circular chromosome [68], we did detect a discreet path of juxtaposition with rightward extension across a diminishing gradient (Fig 7). This path of juxtaposition was eliminated if condensin recruitment was blocked (*fob1Δ* or *lrs4Δ*) or weakened by Brn1depletion with anchor-away, and also consistent with one-sided loop extrusion activity reported for yeast condensin *in vitro* [2]. Similarly, the condensin dosage compensation complex in *C*. *elegans* was recently shown to unidirectionally spread from specific Rex recruitment sites to form loops *in vivo* [69]. On the other hand, human condensin I or II was capable of bidirectional and reversible loop extrusion *in vitro* [70]. Computational models support a requirement for two-sided loop extrusion to compact the much larger vertebrate genomes [71], but single molecule studies in *Xenopus* extracts indicated that loop extrusion by condensin in metaphase was primarily unidirectional, with a smaller fraction bi-directional [72]. Our Micro-C XL results are consistent with the unidirectional model for yeast condensin on chrIII, but do not rule out movement in both directions because *RDT1* lies too close to *HML* and the left telomere to detect extrusion in that direction.

Loop extrusion anchored at the *RDT1* promoter would provide a mechanism that allows *HML* to scan rightward across the right arm of chrIII (Fig 7F), thus increasing the chance of contact with *MAT*a when mating-type switching becomes induced. Given that most donor preference activity comes from the left side of the RE, a domain that shows little effect on chrIII structure [31], we hypothesize the Fob1-dependent chrIII structure has an additional function, perhaps related to regulation of chromosome segregation during mitosis, similar to the rDNA on chrXII. In conclusion, we have established the *RDT1* promoter region of the RE as a new non-repetitive genetic system for future study of condensin loading mechanisms, its regulation by transcription, and programmed structural organization via loop extrusion in yeast.

## Materials and methods

### Yeast strains and plasmids

Yeast strains listed in S2 Table were grown in yeast extract-peptone-dextrose (YPD) or appropriate synthetic complete (SC) dropout medium at 30°C. Gene deletions were constructed by PCR-mediated one-step replacement with *kanMX4*, *natMX6* or *hygMX6* cassettes. C-terminal epitope-tagging of endogenous genes was achieved by integrating 3xHA-*TRP*, 3xV5-*kanMX6*, or 13xMyc-*kanMX6* in place of the stop codon. To generate yeast strains expressing GST-Fob1, a *GST-FOB1* ORF segment was PCR amplified from pJSS92-8 (pGEX6P1 expressing GST-Fob1) and subcloned into *Hin*dIII/*Xba*I sites of pYES2.URA using the In-Fusion method (Takara). The *rdnΔΔ* yeast strain NOY891carrying pNOY353 was maintained on SC-trp 2% galactose [45]. Primers used in strain construction are listed in S3 Table.

### Immunoblots and Immunoprecipitation

Samples for immunoblot analysis were prepared from whole cell extracts using the TCA/glass bead method as previously described [36]. Immunoblots were performed with the following antibodies: anti-myc (Cell Signaling, #71D101:2,000), anti-HA (Cell Signaling, #C29F41:2000), M2 anti-FLAG (Sigma, #F1804, 1:2000), anti-Sir2 (Santa Cruz, 1:2000), or anti-tubulin (Millipore Sigma, #T5168 1:4000). Secondary antibodies were HRP-conjugated anti-mouse (Cell Signaling #7076S, 1:4000) or HRP-conjugated anti-rabbit (Cell Signaling #7074S, 1:4000). Protein–antibody conjugates were revealed by chemiluminescence (Immobilon Western Chemiluminescent HRP substrate, Millipore). To disrupt DNA-mediated protein interactions, cell lysates were digested with Dnase I (100 μg/ml) at 37°C for 20 min prior to antibody incubation.

For immunoprecipitations, yeast strain MD73 (Lrs4-3xHA, Brn1-13x-myc) was grown to OD_600_ of 0.6–0.8, washed, and frozen at -80°C. Cell pellets were resuspended in lysis buffer (50 mM HEPES-KOH [pH 7.5], 150 mM NaCl, 1 mM EDTA, 10% glycerol, 0.5% Nonidet P-40) containing Complete Protease Inhibitor Cocktail (Roche) and then disrupted with glass beads in a Mini-Beader-16 (Biospec Products). Extracts were centrifuged at 13,000 rpm in a microfuge for 15 min at 4°C. Supernatants were incubated with anti-HA ((#C29F41) or anti-myc (9E10) antibody at 4°C for 2 hr, followed by 30 μl of pre-washed Protein A agarose beads (GE) for 2 hr. Beads were collected by centrifugation at 500xg for 5 min, washed twice with wash buffer (50 mM HEPES-KOH [pH 7.5], 150 mM NaCl, 1 mM EDTA), boiled in 1X Laemmli buffer at 95°C for 5 min and run on SDS-PAGE gel for immunoblotting.

### Chromatin immunoprecipitation

ChIP assays were performed as previously described [36]. Recovered DNA was analyzed by real-time PCR using SensiMix SYBR Hi-ROX kit (Bioline, # QT605-05) on a Step One plus real-time PCR machine (Applied Biosystems). PCR primer sequences used for the study are listed in S3 Table. Enrichment was calculated at the respective loci and plotted as percent input. For ChIP-seq experiments, libraries were prepared from the recovered DNA using an Illumina Trueseq ChIP Sample Prep kit (#IP-202-1024) and TrueSeq standard protocol. Single-end sequencing was completed on an Illumina NextSeq 500 at the UVA Genomic Analysis and Technology Core, RRID:SCR_018883. Biological duplicate fastq files for each sample were merged and reads were aligned to the sacCer3 reference genome (release R64-2-1) using Bowtie2 with the following options:—best,—stratum,—nomaqround, and—m10 [73]. The aligned reads were filtered and indexed using SAMtools to generate bam files, which were then converted to bed files using BEDTools [74]. The raw and processed datasets used in this study have been submitted to the NCBI GEO database. The ML1 input replicates are available at GSE92714 (samples GSM2436227 and GSM2436228), and all other ChIP-seq replicates are available at GSE198162. MACS2 was used to call peaks with the following options:—broad,—keep-dup, -tz 150, and -m 3, 1000 [75]. Ctf3 peaks in the WT backgrounds were subtracted from the WT Brn1-13xMyc, WT Lrs4-13xMyc and *lrs4Δ* Brn1-13xMyc peaks, respectively, using BEDTools “intersect” with the–v option. DiffBind was used to call for peaks that were differentially bound in WT Brn1-13xMyc and *lrs4Δ* Brn1-13xMyc samples.

### ChIP-reChIP

ChIP-reChIP was performed as previously described [76]. The steps were similar to standard ChIP with the first primary antibody until elution of the chromatin DNA from beads, which was done with 75 μl reChIP elution buffer (Tris-EDTA, 1% SDS, 10 mM DTT, protease inhibitor cocktail) by incubating at 37°C for 30 min. The eluent was transferred to a new microfuge tube and diluted 20 times with dilution buffer (20 mM Tris HCl, pH 8, 150 mM NaCl, 1% Triton X-100, 2 mM EDTA, protease inhibitor cocktail). The second primary antibody was then added, followed by incubation with Protein G magnetic beads (Thermo Fischer Scientific). Following washes and recovery of DNA with final elution buffer (1% SDS and 100 mM NaHCO_3_), the purified DNA was subjected to qPCR for detecting relative enrichment of co-bound proteins at the indicated loci.

### Micro-C XL

Micro-C XL was performed as previously described [49], with modifications. WT, *lrs4Δ* and *fob1Δ* strains were grown in duplicate or triplicate 100 ml YPD cultures to mid-log phase, then crosslinked with 3% formaldehyde for 10 min at 30°C and quenched with glycine. Cells were centrifuged, washed with sterile water, resuspended in 10 ml Buffer Z (1M sorbitol, 50 mM Tris, pH 7.4) and 10 mM β-Mercaptoethanol, then spheroplasted by adding 250 μl of 20T 10 mg/ml Zymolyase for 1 hr at 30°C. Cell pellets were washed in 1X PBS and crosslinked with 3 mM EGS (ethylene glycol bis(succinimidyl succinate)) for 40 min at 30°C, quenched by addition of glycine, washed, and stored at -80°C. The cell pellets were resuspended in 50 μl of Mbuffer1 and digested with an appropriate amount of MNase (Worthington, #LS004798) to obtain >95% mononucleosomes. The chromatin pellet from four combined reactions was then used for Micro-C XL following the published protocol [49]. Libraries of the recovered ligated dinucleosomal DNA were prepared using NEBNext Ultra II kit (#E7645S, New England Biolabs). Paired-end sequencing was performed on an Illumina NextSeq 500 or NextSeq 2000 at the UVA Genome Analysis and Technology Core, RRID:SCR_018883. Micro-C XL datasets are available at GEO accession number GSE198162.

### Micro-C XL and 3C-seq data analysis

Micro-C XL raw data for each replicate was filtered and mapped into binned and iteratively corrected genome matrix files using the HiC-Pro pipeline [77] on UVA’s high-performance computing Rivanna cluster. Matrix file outputs were further analyzed with the HiTC package in R studio to create the genome and individual chromosome interaction maps presented in this work [78]. A custom awk script was used to parse out the top 10% of annotated genomic interactions presented (S3A and S3B Fig). A custom awk script and the HiTC package was also used to generate subtraction plots for Micro-C XL data and 3C-seq data from a previously reported Brn1 anchor-away experiment with asynchronously grown cells, GEO accession GSE106104 [53]. The control (not depleted) dataset replicates were GSM282946 and GSM282948, while the rapamycin-treated (Brn1-depleted) replicates were GSM282947 and GSM282949. Interaction maps (iteratively corrected or subtraction) for each replicate are provided (S4 Fig). Pooled sequencing data was used for the main figures to maximize resolution. BART3D was used to calculate Differential Chromatin Interaction (DCI scores). The custom awk scripts are available upon request.

### Mating-type switching and donor preference assays

The donor preference assay was performed as previously described [48]. The mating-type switching time course with strains carrying pGAL-HO-URA3 was performed by isolating genomic DNA from cells harvested at each timepoint and the switching detected by relative abundance of *MAT*a and *MAT*α cassettes detected by PCR [27].

### GST-Fob1p purification

Yeast strains were cultured in 2L SC-ura with 2% raffinose to log phase, then centrifuged at 3500 rpm in a F9-4x1000Y rotor (Piramoon Technologies) for 15 min at room temperature. Cells were resuspended and incubated in SC-ura with 2% galactose to induce GST-Fob1 expression for 4 hrs. Following centrifugation and washing, cell pellets were resuspended in 20 ml lysis buffer (50 mM HEPES, pH 7.6, 10% glycerol, 10 mM EDTA, 0.5M NaCl, 1% Triton X-100, 5 mM DTT, 1X protease inhibitor cocktail, 1 mM PMSF) and disrupted with glass beads using a BeadBeater (Biospec Products). The lysate was centrifuged at 15000 rpm in a Sorvall SS-34 rotor for 10 min at 4°C and the soluble extracts diluted with lysis buffer to a final NaCl concentration of 0.1M, then incubated with 2 ml glutathione 4B-sepharose (GE, 17-0756-01) for 4 hr at 4°C. The resin was washed 2 times with wash buffer (50 mM HEPES, pH 7.6, 10% glycerol, 10 mM EDTA, 0.1M NaCl, 1% Triton X-100, 5 mM DTT, 1X protease inhibitor, 1 mM PMSF) and the GST fusion protein was eluted with 400 μl elution buffer (50 mM HEPES, pH 7.6, 10% glycerol, 100 mM NaCl, 1mM DTT, 10 mM glutathione). The eluate was concentrated in a 3K MWCO filter Amicon Ultra centrifugal filter at 14000 g for 10 min at 4°C. Isolation of the fusion protein was confirmed by immunoblot with α-Fob1 antibody (Santa Cruz, sc-98575, 1:1000 dilution).

### EMSA

DNA fragments for EMSA were PCR amplified from genomic DNA with 5’-biotinylated primers (S3 Table), then purified from a 12% polyacrylamide gel. EMSA was performed with a LightShift Chemiluminescent EMSA kit (Thermo Fischer, 20148). 5 ng of probe was incubated with 60 ng GST or GST- Fob1p in 20 μl 1X buffer (10 mM Tris, pH 7.5, 1 mM EDTA, 0.1M KCl, 0.1mM DTT, 5% glycerol) and incubated at 30°C for 30 min. As competitor, 50 ng or 200 ng unlabeled probe was added. The RFB fragment was used a positive control for direct interaction [10], while a nuclear extract supplied with the kit was used as a positive gel shift control. Samples were run on a 6% polyacrylamide gel in 0.5X TBE, transferred to Immobilon-Ny^+^ at 380mA (~100V) for 30 minutes using mini Trans-blot electrophoretic system (Bio-Rad) and autocrosslinked at 120 mJ/cm^2^ in a Stratagene Stratalinker 1800. The biotin-labeled DNA was detected by using LightShift Chemiluminescent EMSA reagents according to the manufacturer’s instructions. Two independent EMSA experiments were performed and the level of specific binding competition with unlabeled probes quantitated (Figs 4D and S2). ImageJ was used for quantifying band intensity.

## Supporting information

S1 Fig ChIP-seq enrichment peaks for Lrs4-myc and Brn1-myc at the rDNA locus. (**A**) IGV snapshot for chromosome XII of ChIP-seq data showing the enrichment of Brn1-myc and Lrs4-myc, and loss of Brn1-myc binding in the *lrs4Δ* mutant. Ctf3-myc is used as a control for non-specific binding independent of centromeres. Tracks were normalized to total read count and comprise the average enrichment across all rDNA repeats. (**B**) ChIP assay with Lrs4-myc showing lack of enrichment on the *RDT1* open reading frame in *MAT*a and *MAT*α cells. Assay was run in biological triplicates with standard deviations calculated.
(TIF)

S2 Fig Quantitation of Fob1 EMSA assay. (**A**) Duplicate EMSA assay recapitulating the representative results reported in Fig 4D. (**B**) Quantitation of bandshift competition from the unlabeled *RDT1* or RFB probes. Band shifts 1 and 2, and the supershift are indicated by asterisks and arrows. Bandshift intensity without any competitor probe was normalized to 100%.
(TIF)

S3 Fig Differential Chromatin Interaction (DCI) analysis of *lrs4Δ* and *fob1Δ* mutants. (**A-B**) Genomic interactions between centromeres (**A**) and telomeres (**B**) across 10 kb bins in *lrs4*Δ and *fob1*Δ. p < 0.05 calculated using a difference of means permutation test of *CEN/TEL* bins versus the rest of the genome. (**C-D**) Distribution of DCI scores for all 5 kb bins in the genome for *lrs4*Δ (**C**) and *fob1*Δ (**D**) compared to Wild Type. (**E-F***)* Ranked list of 5 kb bins with the strongest negative DCI scores. The 5kb bin containing *RDT1* highlighted in red. Analysis was performed with pooled reads from replicate samples.
(TIF)

S4 Fig Individual replicate comparisons of Micro-C XL and 3C-seq interaction heatmaps. (**A-C**) Triplicate WT vs. *lrs4Δ* iteratively corrected contact map comparisons. (**D-F**) Triplicate WT minus *lrs4Δ* interaction subtraction plots. (**G-H**) Duplicate WT vs. *fob1Δ* iteratively corrected contact map comparisons. (**I-J**) Duplicate WT minus *fob1Δ* interaction subtraction plots. Note the consistent loss of *RDT1*-anchored (bin 4) contact across the right arm of chrIII. The color scales indicate maximum and minimum interaction counts from 10kb intervals across the genome.
(TIF)

S5 Fig Micro-C XL and 3C-seq analysis of chrXII conformation. (**A**) Schematic of condensin, cohibin, Tof2, and RENT recruitment to the IGS1 region of rDNA repeats through interactions with Fob1 bound to its TER1 and TER2 binding sites. A TAD-like region between *CEN12* and the rDNA on chrXII is also depicted. (**B-C**) Micro-C XL subtraction plots of chrXII for *lrs4Δ* and *fob1Δ* compared to WT at 10kb resolution. (**D**) 3C-seq subtraction plot for acute Brn1 depletion by anchor-away compared to without depletion. Red shading indicates higher contact frequency in WT and blue shading indicates higher contract frequency in the mutant condition. Subtraction values in panels B-D are arbitrarily scaled to be in the linear color scale range.
(TIF)

S1 Table Excel spreadsheet with Brn1-myc ChIP-seq binding sites showing significantly different enrichment in WT compared to *lrs4Δ* strains. (XLSX)

S2 Table List of yeast strains used in the study. (PDF)

S3 Table List of oligonucleotides used in the study. (PDF)
