# Supplementary figures and images for: Fob1-dependent condensin recruitment and loop extrusion on yeast chromosome III
Source: PLoS Genet. 2023 Apr 14;19(4):e1010705. doi: 10.1371/journal.pgen.1010705 (PMC10132618; doi:10.1371/journal.pgen.1010705)

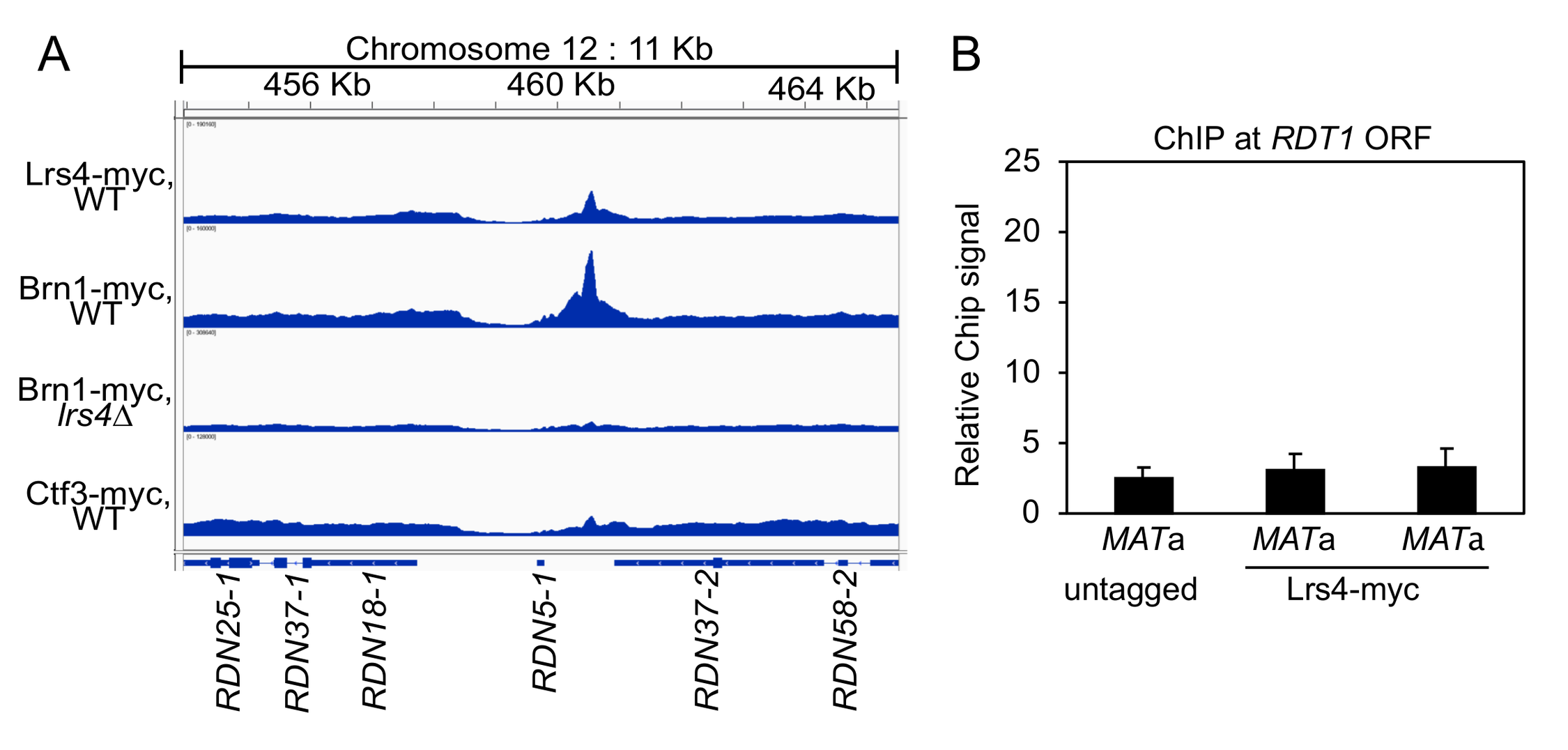

Supplement: S1 Fig — (A) IGV snapshot for chromosome XII of ChIP-seq data showing the enrichment of Brn1-myc and Lrs4-myc, and loss of Brn1-myc binding in the lrs4Δ mutant. Ctf3-myc is used as a control for non-specific binding independent of centromeres. Tracks were normalized to total read count and comprise the average enrichment across all rDNA repeats. (B) ChIP assay with Lrs4-myc showing lack of enrichment on the RDT1 open reading frame in MATa and MATα cells. Assay was run in biological triplicates with standard deviations calculated. (TIF) [file pgen.1010705.s001.tif]

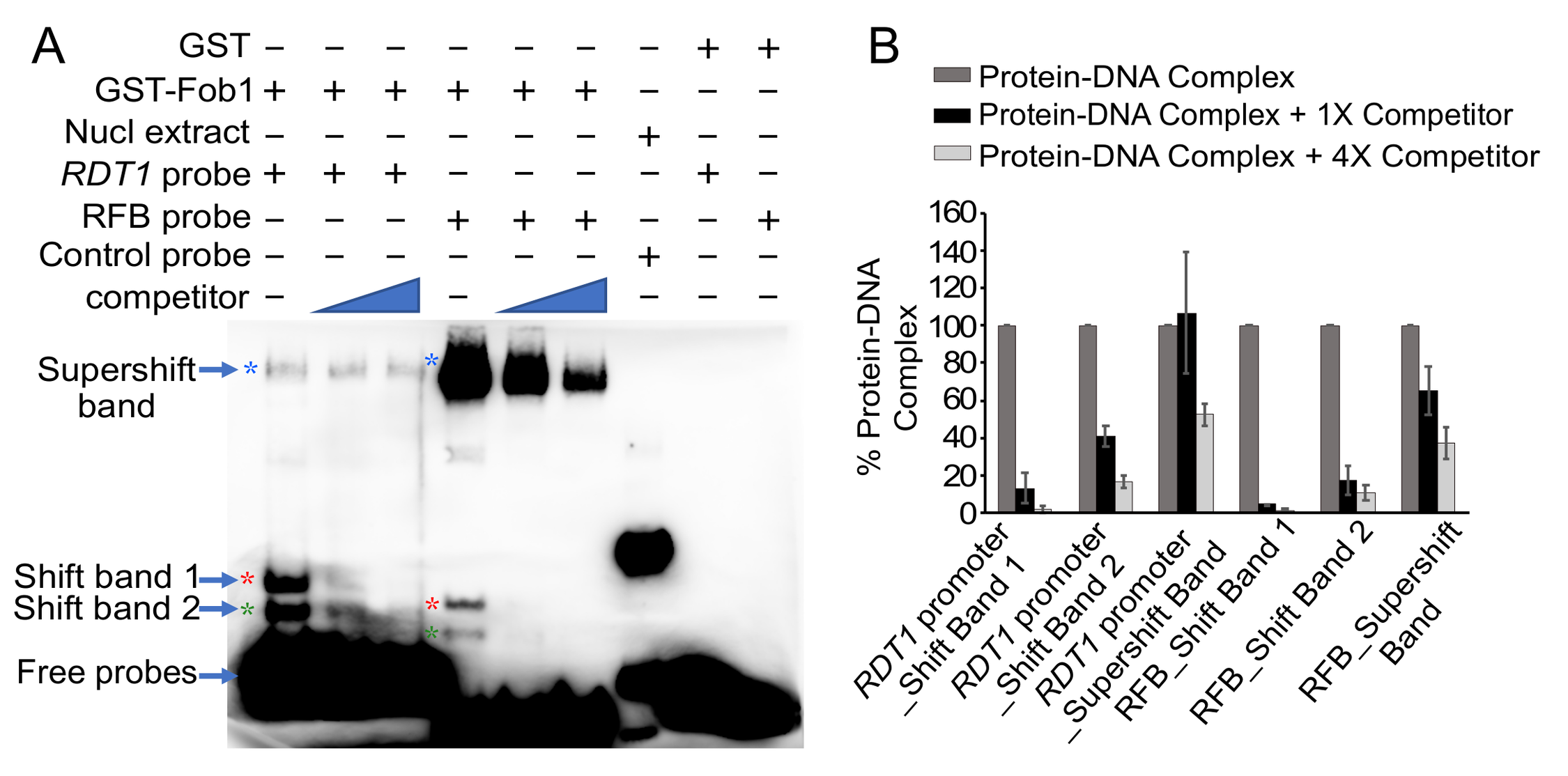

Supplement: S2 Fig — (A) Duplicate EMSA assay recapitulating the representative results reported in Fig 4D. (B) Quantitation of bandshift competition from the unlabeled RDT1 or RFB probes. Band shifts 1 and 2, and the supershift are indicated by asterisks and arrows. Bandshift intensity without any competitor probe was normalized to 100%. (TIF) [file pgen.1010705.s002.tif]

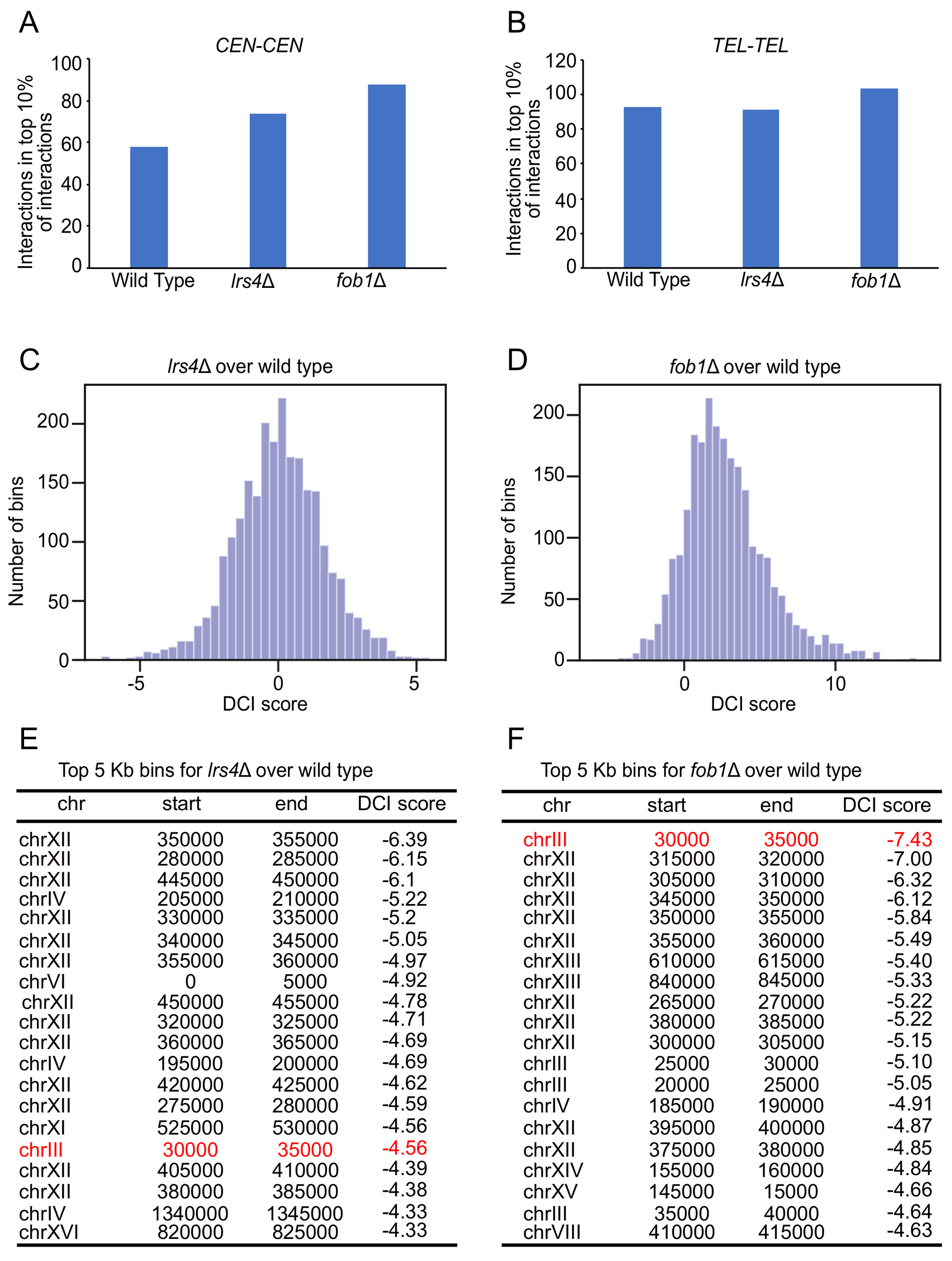

Supplement: S3 Fig — (A-B) Genomic interactions between centromeres (A) and telomeres (B) across 10 kb bins in lrs4Δ and fob1Δ. p < 0.05 calculated using a difference of means permutation test of CEN/TEL bins versus the rest of the genome. (C-D) Distribution of DCI scores for all 5 kb bins in the genome for lrs4Δ (C) and fob1Δ (D) compared to Wild Type. (E-F) Ranked list of 5 kb bins with the strongest negative DCI scores. The 5kb bin containing RDT1 highlighted in red. Analysis was performed with pooled reads from replicate samples. (TIF) [file pgen.1010705.s003.tif]

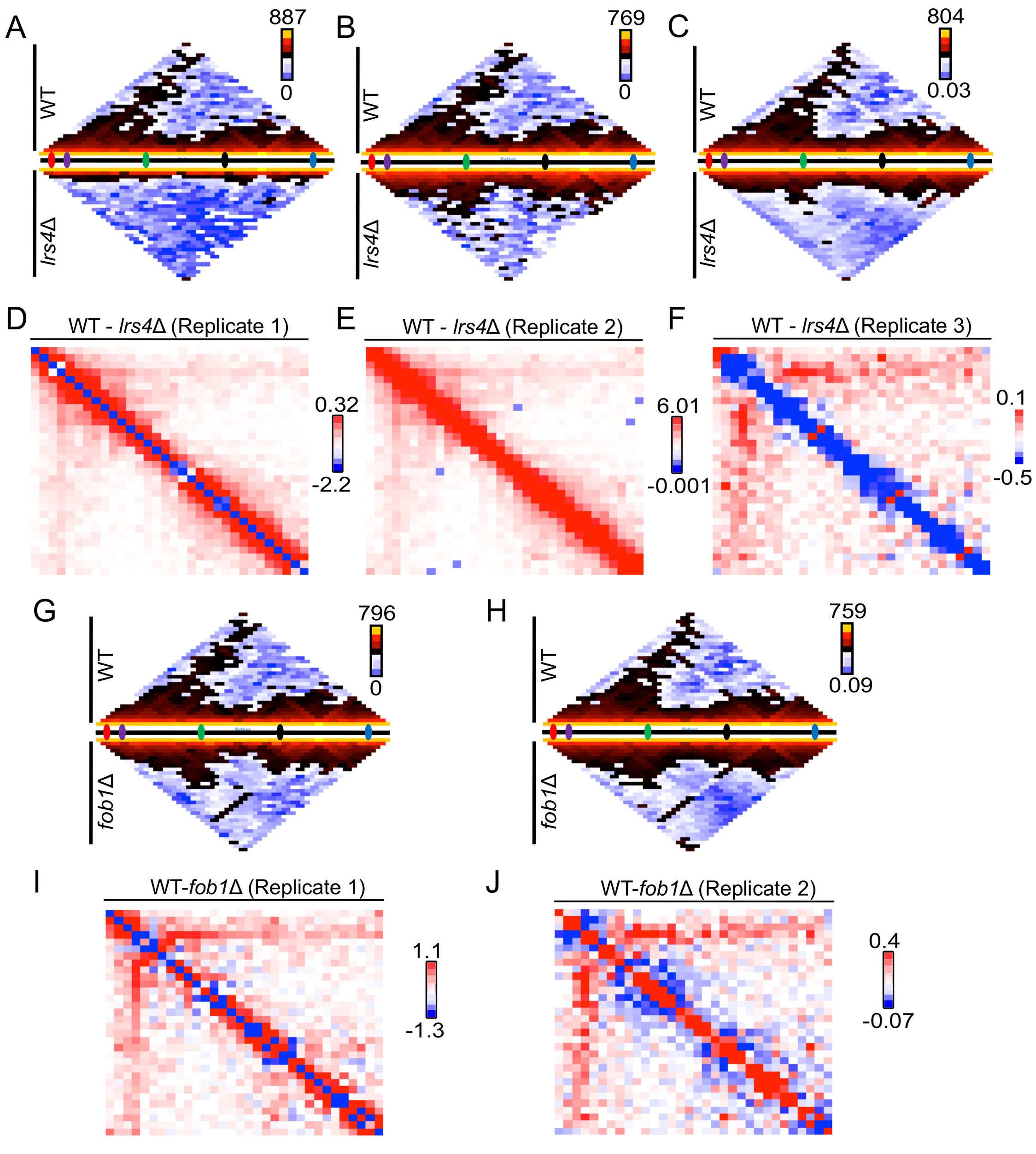

Supplement: S4 Fig — (A-C) Triplicate WT vs. lrs4Δ iteratively corrected contact map comparisons. (D-F) Triplicate WT minus lrs4Δ interaction subtraction plots. (G-H) Duplicate WT vs. fob1Δ iteratively corrected contact map comparisons. (I-J) Duplicate WT minus fob1Δ interaction subtraction plots. Note the consistent loss of RDT1-anchored (bin 4) contact across the right arm of chrIII. The color scales indicate maximum and minimum interaction counts from 10kb intervals across the genome. (TIF) [file pgen.1010705.s004.tif]

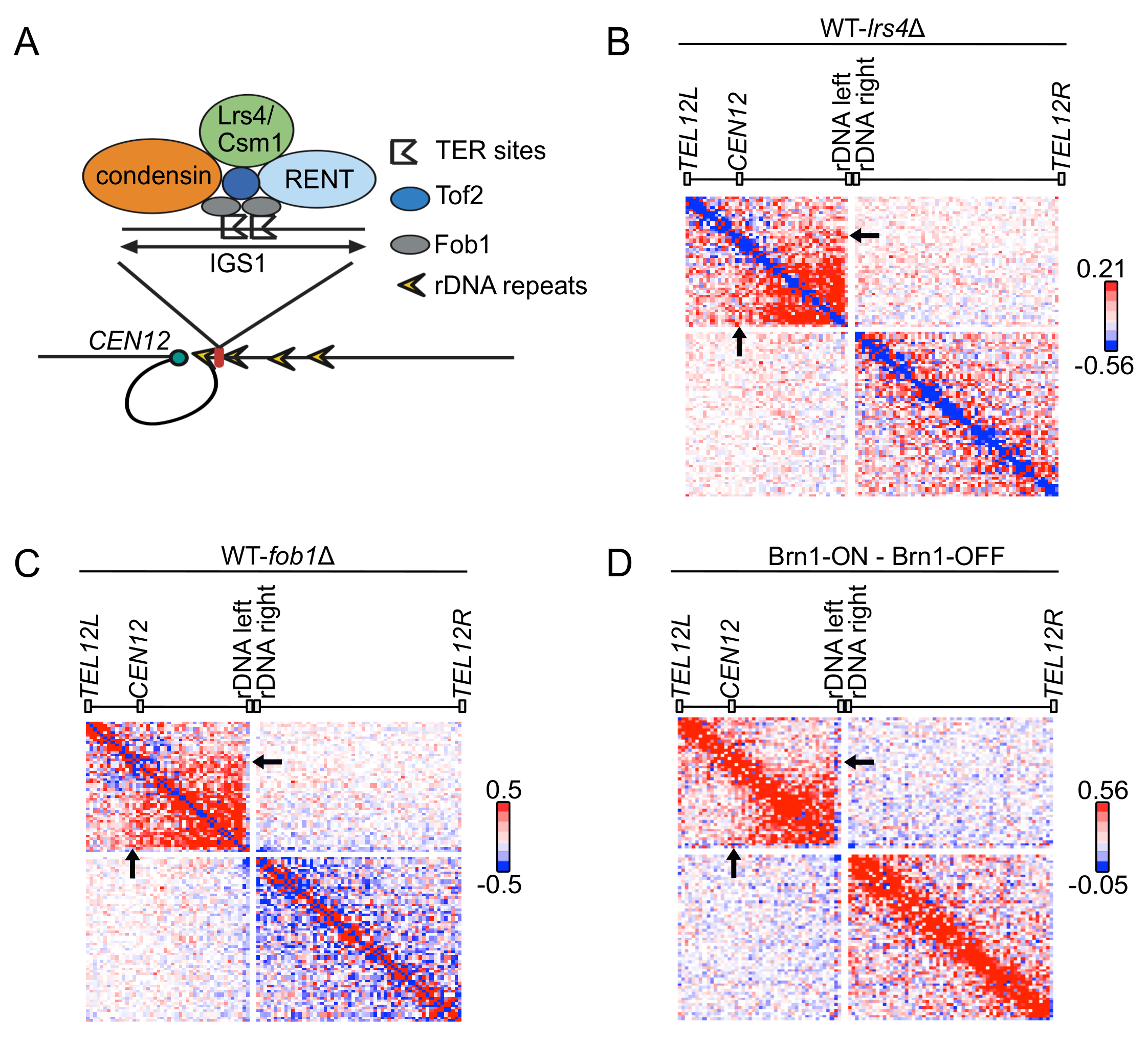

Supplement: S5 Fig — (A) Schematic of condensin, cohibin, Tof2, and RENT recruitment to the IGS1 region of rDNA repeats through interactions with Fob1 bound to its TER1 and TER2 binding sites. A TAD-like region between CEN12 and the rDNA on chrXII is also depicted. (B-C) Micro-C XL subtraction plots of chrXII for lrs4Δ and fob1Δ compared to WT at 10kb resolution. (D) 3C-seq subtraction plot for acute Brn1 depletion by anchor-away compared to without depletion. Red shading indicates higher contact frequency in WT and blue shading indicates higher contract frequency in the mutant condition. Subtraction values in panels B-D are arbitrarily scaled to be in the linear color scale range. (TIF) [file pgen.1010705.s005.tif]
