# Supplementary material for: Fob1-dependent condensin recruitment and loop extrusion on yeast chromosome III
Source: PLoS Genet. 2023 Apr 14;19(4):e1010705. doi: 10.1371/journal.pgen.1010705 (PMC10132618; doi:10.1371/journal.pgen.1010705)
Supplement: S2 Table — (PDF) [file pgen.1010705.s007.pdf]

Supplemental Table S2. Yeast Strains

Strains	Genotype	Reference
ML1	*MAT**a** his3Δ200 leu2Δ1 met15Δ0 trp1Δ63 ura3-167*	1
ML25B12	*MATα his3Δ200 leu2Δ1 met15Δ0 trp1Δ63 ura3-167*	2
ML44	*MAT**a*** his3Δ200 *leu2Δ1 met15Δ0 ura3Δ0 trp1Δ63 SIR2-13xMyc-kanMX4*	1
MD99	*MAT**a** his3Δ200 leu2Δ1 met15Δ0 ura3Δ0 trp1Δ63 CTF3 13xMyc::KanMX4*	This study
RF28	*MAT**a** his3Δ200 leu2Δ1 met15Δ0 ura3Δ0 trp1Δ63 FOB1-13xMyc-kanMX4*	This study
MD134	*MAT**a** his3Δ200 leu2Δ1 met15Δ0 ura3Δ0 trp1Δ63 FOB1-13xMyc-kanMX4 dps2Δ*	This study
RF29	*MATα his3Δ200 leu2Δ0 met15Δ0 ura3Δ0 trp1Δ63 FOB1-13xMyc-kanMX4*	This study
ML149	*MAT**a** his3Δ200 leu2Δ1 met15Δ0 trp1Δ63 ura3-167 BRN1-13xMyc-kanMX4*	3
ML465	*MAT**a** his3Δ200 leu2Δ1 met15Δ0 trp1Δ63 ura3-167 LRS4-13xMyc-kanMX4*	This study
ML526	*MAT**a** his3Δ200 leu2Δ1 met15Δ0 trp1Δ63 ura3-167 LRS4-13xMyc-kanMX4 dps2Δ*	This study
ML468	*MAT**a** his3Δ200 leu2Δ1 met15Δ0 trp1Δ63 ura3-167 lrs4Δ::kanMX4*	This study
ML494	*MAT**a** his3Δ200 leu2Δ1 met15Δ0 trp1Δ63 ura3-167 BRN1-13xMyc-kanMX4 lrs4Δ::kanMX4*	This study
ML152	*MAT**a** his3Δ200 leu2Δ1 met15Δ0 trp1Δ63 ura3-167 SMC4-13xMyc-kanMX4*	3
ML160	*MAT**a** his3Δ200 leu2Δ1 met15Δ0 trp1Δ63 ura3-167 SMC4-13xMyc-kanMX4 sir2Δ::natMX4*	3
ML161	*MAT**a** his3Δ200 leu2Δ1 met15Δ0 trp1Δ63 ura3-167 BRN1-13xMyc-kanMX4 sir2Δ::natMX4*	3
SY337	*MATa his3Δ1 leu2Δ0 met15Δ0 ura3Δ0 MCM1-13xmyc-HIS3MX*	4
RF186	*MATa his3Δ1 leu2Δ0 met15Δ0 ura3Δ0 CSM1-5xFlag-hphMX4*	This study
ML506	*MATa his3Δ1 leu2Δ0 met15Δ0 ura3Δ0 MCM1-13xMyc-HIS3MX CSM1-5xFlag-hphMX6*	This study
MD73	*MAT**a** his3Δ200 leu2Δ1 met15Δ0 trp1Δ63 ura3-167 BRN1-13xMyc-kanMX4 LRS4-3xHA-TRP1*	This study
ML440	*MAT**a** his3Δ200 leu2Δ1 met15Δ0 trp1Δ63 ura3-167* [pGAL-HO-*URA3*]	2
MD69	*MAT**a** his3Δ200 leu2Δ1 met15Δ0 trp1Δ63 ura3-167, lrs4Δ::kanMX4* [pGAL-HO-*URA3*]	This study
ML458	*MAT**a** his3Δ200 leu2Δ1 met15Δ0 trp1Δ63 ura3-167 sir2Δ::KanMX4* [pGAL-HO-*URA3*]	2
ML444	*MAT**a** his3Δ200 leu2Δ1 met15Δ0 trp1Δ63 ura3-167 BRN1-13xMyc-kanMX4* [pGAL-HO-*URA3*]	This study
XW652	*MAT**a** ho ade3::GAL::HO HMLα RE HMRα*-B *ura3-52 lys5 leu2-3,112 trp1::hisG*	5
XW676	*MAT**a** ho ade3::GAL::HO HMLα REΔ::URA3 HMRα*-B *ade1 leu2 trp1 ura3-52*	5

SY762	XW652 made *lrs4Δ::kanMX4*	This study
MD135	XW652 made *fob1Δ::kanMX4*	This study
W303a	*MAT***a** *ade2- 1 ura3-1 his3-11,15 trp1-1 leu2-3,112 can1-100*	
W303b	*MATα ade2- 1 ura3-1 his3-11,15 trp1-1 leu2-3,112 can1-100*	
MD89	ML465, *fob1Δ::natMX4*	This study
MD90	ML149, *fob1Δ::natMX4*	This study
MD92	ML465, *tof2Δ::natMX4*	This study
MD93	ML149, *tof2Δ::natMX4*	This study
MD117	*MATa his3Δ1 leu2Δ0 met15Δ0 ura3Δ0* [pYES2.URA.GST-FOB1]	This study
MD113	*MAT***a** *his3Δ200 leu2Δ1 met15Δ0 trp1Δ63 ura3-167 fob1Δ::kanMX4*	This study
NOY891	*MAT***a** ade2-1 *ura3-1 trp1-1 leu2-3,112 his3-11 can1-100 rdnΔΔ::HIS3* [pNOY353]	6
MD115	*MAT***a** ade2-1 *ura3-1 trp1-1 leu2-3,112 his3-11 can1-100 rdnΔΔ::HIS3 BRN1-myc::KanMX* [pNOY353]	This study
MD116	*MAT***a** ade2-1 *ura3-1 trp1-1 leu2-3,112 his3-11 can1-100 rdnΔΔ::HIS3 LRS4-myc::KanMX* [pNOY353]	This study
MD120	*MAT***a** ade2-1 *ura3-1 trp1-1 leu2-3,112 his3-11 can1-100 rdnΔΔ::HIS3 SIR2-myc::KanMX* [pNOY353]	This study
MD121	*MAT***a** ade2-1 *ura3-1 trp1-1 leu2-3,112 his3-11 can1-100 rdnΔΔ::HIS3 FOB1-myc::KanMX* [pNOY353]	This study

References

1. Li, M., Petteys, B.J., McClure, J.M., Valsakumar, V., Bekiranov, S., Frank, E.L., and Smith, J.S. Thiamine biosynthesis in *Saccharomyces cerevisiae* is regulated by the NAD^+-dependent histone deacetylase Hst1. Mol Cell Biol. 2010;30(13):3329-41. PMID: 20439498.

2. Li, M., Fine, R.D., Dinda, M., Bekiranov, S., and Smith, J.S. A Sir2-regulated locus control region in the recombination enhancer of *Saccharomyces cerevisiae* specifies chromosome III structure. PLoS Genet. 2019;15(8): e1008339. PMID: 31461456.

3. Li, M., Valsakumar, V., Poorey, K., Bekiranov, S., and Smith, J.S. Genome-wide analysis of functional sirtuin chromatin targets in yeast. Genome Biol. 2013;14(5): R48. PMID: 23710766.

4. Yoon, S., Govind, C.K., Qiu, H., Kim, S.J., Dong, J., and Hinnebusch, A.G. Recruitment of the ArgR/Mcm1p repressor is stimulated by the activator Gcn4p: a self-checking activation mechanism. Proc Natl Acad Sci U S A. 2004;101(32): 11713-8. PMID: 15289616.

5. Li, J., Coic, E., Lee, K., Lee, C.S., Kim, J.A., Wu, Q., and Haber, J.E. Regulation of budding yeast mating-type switching donor preference by the FHA domain of Fkh1. PLoS Genet. 2012;8(4): e1002630. PMID: 22496671.

6. Wai, H.H., Vu, L., Oakes, M., and Nomura, M. Complete deletion of yeast chromosomal rDNA repeats and integration of a new rDNA repeat: use of rDNA deletion strains for functional analysis of rDNA promoter elements *in vivo*. Nucleic Acids Res. 2000;28(18): 3524-34. PMID: 10982872.
