# Supplementary material for: Fob1-dependent condensin recruitment and loop extrusion on yeast chromosome III
Source: PLoS Genet. 2023 Apr 14;19(4):e1010705. doi: 10.1371/journal.pgen.1010705 (PMC10132618; doi:10.1371/journal.pgen.1010705)
Supplement: S3 Table — (PDF) [file pgen.1010705.s008.pdf]

Supplemental Table S3. Oligonucleotides

Name	Description	DNA sequence
JS301	*MAT*a / *MAT*α PCR primer (FW)	AGTCACATCAAGATCGTTTATGG
JS302	*MAT*α specific PCR primer (RV)	GCACGGAATATGGGACTACTTCG
JS854	*MAT*a specific PCR primer (RV)	ACTCCACTTCAAGTAAGAGTTTC
JS467	*kanMX* 5'-out detection	TACGGGCGACAGTCACATCATG
JS1830	*SCR1* PCR primer (FW)	AATGGCTTTCTGGTGGGATG
JS1831	*SCR1* PCR primer (RV)	TTGTTCCTCGGCCAGAATTC
JS2791	*CSM1*-3xFlag (FW)	AAGCCTGAATAAGAAAAGAGAGAAAAAAGATGAA ACCGAG CGGATCCCCGGGTTAATTAA
JS2792	*CSM1*-3xFlag (RV)	CGGAATTTTATGTGTAGATATATAGACATTATGTA GCAGC GAATTCGAGCTCGTTTAAAC
JS2787	*MCM1*-13xMyc (FW)	CTACCAACAATACTTTCAAGAACCGCAACAAGGC CAATACCGGATCCCCGGGTTAATTAA
JS2788	*MCM1*-13xMyc (RV)	TTAATGCTCGTCTATGAATTATATACGGAAATCGA TAAGAGAATTCGAGCTCGTTTAAAC
JS2127	*BRN1*-13xMyc (FW)	AGTGAATTATGAGGATCTAGCGACAACACAGGC AGCGTCACGGATCCCCGGGTTAATTAA
JS2128	*BRN1*-13xMyc (RV)	GCACAAAAAAAAAAAAAAAAAAAAAAAAAAAAGAT CATCAAGAATTCGAGCTCGTTTAAAC
JS2134	*SMC4*-13xMyc (FW)	AAGTACCACGATTAAAAACATAGATATCTTAAACA GAACTCGGATCCCCGGGTTAATTAA
JS2135	*SMC4*-13xMyc (RV)	TTACAATCAGCAAGTGCTCTTGAATTGATTATTGT ACTAGGAATTCGAGCTCGTTTAAAC
JS2789	*LRS4*-13xMyc (FW)	TGAAGAATTGAGTAATAATTTAAATGTTGACGAGT TTGTA CGGATCCCCGGGTTAATTAA
JS2790	*LRS4*-13xMyc (RV)	AAAAGAGAGAGGGAAGGGCAGGGACGTAGATA GCTGTTAC GAATTCGAGCTCGTTTAAAC
JS1191	*SIR2*-13xMyc (FW)	CGTGTATGTCGTTACATCAGATGAACATCCCAAA ACCCTCCGGATCCCCGGGTTAATTAA
JS1192	*SIR2*-13xMyc (RV)	TATTAATTTGGCACTTTTAAATTATTAAATTGCCTT CTACGAATTCGAGCTCGTTTAAAC
JS3273	*CTF3*-13xMyc (FW)	ATATTTGAAGAGTCTCTCAAAGTATAGCGTTCAAA ATTCTCGGATCCCCGGGTTAATTAA
JS3274	*CTF3*-13xMyc (RV)	TATATATATATATATATGTATATAAATGTGGATGC ATATTGAATTCGAGCTCGTTTAAAC
JS3266	*FOB1*-13xMyc (FW)	TGGAGATCAAACAAGAGACTTTGGCACATCAATG GAATTGCGGATCCCCGGGTTAATTAA
JS3267	*FOB1*-13xMyc (RV)	ACCTATGGTGACTCCTCCTTTCATTCTATCCTACA TATTAGAATTCGAGCTCGTTTAAAC
JS3191	*LRS4*-3xHA (FW)	TGAAGAATTGAGTAATAATTTAAATGTTGACGAGT TTGTACGGATCCCCGGGTTAATTAAC
JS2790	*LRS4*-3xHA (RV)	AAAAGAGAGAGGGAAGGGCAGGGACGTAGATA GCTGTTAC GAATTCGAGCTCGTTTAAAC
JS2858	*rdt1*Δ using pCORE (FW)	CGAAAACTGCAGCATGTATTTAATCGAAAACTAC AGCATGTAGCTAGGGTATTGAAAAATTTCTAGAA CCAGAAATAATCGAAAGCATTA
JS2859	*rdt1*Δ using pCORE (RV)	TAATGCTTTCGATTATTTCTGGTTCTAGAAATTTT

		TCAATACCCTAGCTACATGCTGTAGTTTTCGATTA AATACATGCTGCAGTTTTCG
JS2444	pCORE 100bpΔ replacement (FW)	GACTTACAAGCACACCTTTGAATTATTTTTGTTCT CTATGACTTATAAAATGAATATTTCAATTGATGAA TAGCTATATA
JS2445	pCORE 100bpΔ replacement (RV)	TATATAGCTATTCATCAATTGAAATATTCATTTTAT AAGTCATAGAGAACAAAAATAATTCAAAGGTGTG CTTGTAAGTC
JS3073	*RDT1* promoter ChIP as well as *RDT1*_unlabelled_EMSA (FW)	TGCTAGTGTTTGCAAGATGGTGC
JS3074	*RDT1* promoter ChIP as well as RDT1_unlabelled_EMSA (RV)	AGGAGCAGAAACGTGGCAAT
JS1100	*NTS1* ChIP (FW)	TGTTAGTGCAGGAAAGCGGG
JS1101	*NTS1* ChIP (RV)	CTACACCCTCGTTTAGTTGC
JS3044	*lrs4Δ natMX* (FW)	GGTTATTAAACATTTCTATTAGGTGCTTCTTCTTT GCCGC GATTGTACTGAGAGTGCACC
JS3045	*lrs4Δ natMX* (RV)	AAAAGAGAGAGGGAAGGGCAGGGACGTAGATA GCTGTTAC CTGTGCGGTATTTC ACACCG
JS3257	*tof2Δ natMX* (FW)	ATATCCTTTGCCCTCCTTGGTGTTTGGTTCAAATA AGTAACGGCGCGAAGCAAAAATTACGGC
JS3258	*tof2Δ natMX* (RV)	AAGATATGGTTGAGAGATCCCAACATAATTACTG ACAGACCGGCGTTAGTATCGAATCGACAGC
JS900	*fob1Δ natMX* (FW)	GGAGAACAATTTAACGATTGTGTGAGTGTGAATT TGTGCT CGGCGCGAAGCAAAAATTACGGC
JS901	*fob1Δ natMX* (RV)	CAC CTA TGA CTC CTC CTT TCA TTC TAT CCT ACA TAT TCG GCG TTA GTA TCG AAT CGA CAG C
JS2665	*SCR1* ORF (FW)	CGTTGAGAATTCTGGCCGAG
JS2666	*SCR1* ORF (RV)	GTAAATCCTGATGGCACCGC
JS2669	KanC3	CCTCGACATCATCTGCCCAGAT
JS3301	GST-Fob1_infusion_HindIII (FW)	CTATAGGGAATATTAAGCTTATGTCCCCTATACTA GGTTATTGG
JS3302	GST-Fob1_infusion_XbaI (RV)	ACATGATGCGGCCCTCTAGATTACAATTCCATTG ATGTGCCAAAG
JS3305	100bp_labelled_EMSA (FW)	biotin-TGCTAGTGTTTGCAAGATGGTGC
JS3306	RFB_labelled_EMSA (FW)	biotin-ATTCTCTAAACTTATACAAGCACT
JS3307	RFB_unlabelled_EMSA (FW)	ATTCTCTAAACTTATACAAGCACT
JS3308	RFB_unlabelled_EMSA (RV)	ATCAGAGCGGCAAACATG
